# Biomimetic bilayer hydrogel coating with antithrombotic and anticalcification properties for cardiovascular tissue engineering application

**DOI:** 10.1093/rb/rbaf122

**Published:** 2025-12-01

**Authors:** Shisong Chen, Qin Li, Chao Ye, Jiajun Pan, Si Chen, Jingwen Zhou, Lei Liu, Jiajun Zhang, Zhiyun Xu, Bailing Li, Lin Han, Xiaohong Liu

**Affiliations:** Department of Cardiovascular Surgery Laboratory, Changhai Hospital, Navy Medical University, Shanghai 200433, China; Department of Cardiovascular Surgery Laboratory, Changhai Hospital, Navy Medical University, Shanghai 200433, China; Department of Cardiovascular Surgery Laboratory, Changhai Hospital, Navy Medical University, Shanghai 200433, China; Department of Cardiovascular Surgery, Changhai Hospital, Navy Medical University, Shanghai 200433, China; Department of Cardiovascular Surgery, Changhai Hospital, Navy Medical University, Shanghai 200433, China; Department of Cardiovascular Surgery Laboratory, Changhai Hospital, Navy Medical University, Shanghai 200433, China; Department of Cardiovascular Surgery Laboratory, Changhai Hospital, Navy Medical University, Shanghai 200433, China; Department of Cardiovascular Surgery, Changhai Hospital, Navy Medical University, Shanghai 200433, China; Department of Cardiovascular Surgery, Changhai Hospital, Navy Medical University, Shanghai 200433, China; Department of Cardiovascular Surgery, Changhai Hospital, Navy Medical University, Shanghai 200433, China; Department of Cardiovascular Surgery, Changhai Hospital, Navy Medical University, Shanghai 200433, China; Department of Cardiovascular Surgery Laboratory, Changhai Hospital, Navy Medical University, Shanghai 200433, China

**Keywords:** cardiovascular application, tissue engineering, decellularized extracellular matrix, anticalcification, antithrombosis

## Abstract

Decellularized extracellular matrix (dECM), a promising tissue engineering scaffold for cardiovascular applications, might exhibit enhanced durability when endowed with anticalcification and antithrombotic properties. Herein, we present a biomimetic bilayer hydrogel coating applied to acellular swim bladders (ASBs). First, we designed an endothelium-mimicking (HCT) hydrogel coating, comprising alternately assembled endothelial glycocalyx macromolecule hyaluronic acid, copper ions, and tannic acid. Subsequently, a hydrophilic methacrylated silk fibroin (SilMA) hydrogel was incorporated as the outer coating layer. Notably, the HCT hydrogel penetrated and anchored into the ASB matrix, forming an interpenetrating network that enhanced the biostability and mechanical properties of the ASB matrix. Additionally, the SilMA hydrogel enhanced the hydrophilicity and antifouling properties of the HCT coating. *In vitro* experiments and subcutaneous implantation further revealed that the bilayer hydrogel (H/S) coating exhibited excellent biocompatibility, hemocompatibility, antibacterial activity, and anticalcification properties. Furthermore, a blood circulation model and rabbit shunt assay confirmed the great anticoagulation properties of the H/S coating. Moreover, in an *in vivo* rat carotid aorta replacement model, the H/S coating effectively promoted endothelialization, enhanced vascular remodeling, prevented calcification and thrombosis, and ultimately improved ASB durability. Based on these findings, our endothelium-mimicking hydrophilic bilayer hydrogel coating holds great promise as a surface modification strategy for tissue engineering scaffolds.

## Graphical abstract

**Figure:**
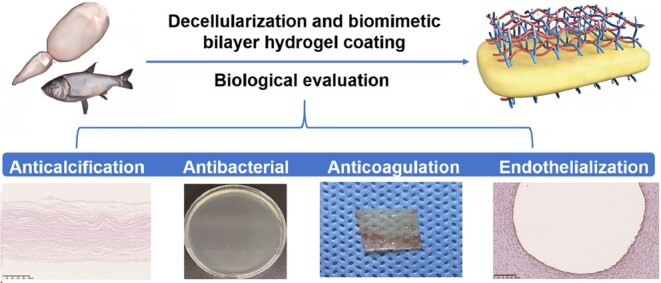

## Introduction

Cardiovascular lesions, including structural cardiac diseases, arterial diseases, and trauma, are among the leading causes of mortality worldwide, making them a major public health concern [1]. Surgical interventions are necessary to repair or replace the damaged tissues with synthetic or glutaraldehyde (Glut)-crosslinked biological materials. Despite their usefulness in the surgical therapy of cardiovascular lesions, these materials have been associated with certain limitations. For example, synthetic materials are highly susceptible to infection [2], while Glut-crosslinked biomaterials are prone to calcification [3], with both also having poor biocompatibility, lacking tissue growth capacity, and presenting a high risk of thrombosis [4, 5]. Therefore, developing more durable and biocompatible materials with enhanced anti-infection, anticalcification, and antithrombosis properties would be imperative for improved outcomes.

Decellularized extracellular matrix (ECM), a natural scaffold material with good biocompatibility and bioactive properties, is widely utilized in tissue engineering [6, 7]. Although decellularization could eliminate xenogeneic antigens and calcium influx to reduce calcification, the associated functional component loss and collagen fiber exposure could trigger coagulation, as well as platelet activation and aggregation, leading to thrombosis [8], which in turn initiates calcification.

Notably, hydrogel coating could improve the functional properties of decellularized ECM. Hyaluronic acid (HA), a glycosaminoglycan (GAG) present in the ECM and endothelial glycocalyx, could provide a mechanical and physiological environment for tissue hemostasis. Therefore, exogenous HA supplementation might accelerate the endothelialization of blood-contacting biomaterials and suppress both thrombosis and calcification [9]. Although HA hydrogels have been used in various biomaterials for tissue engineering applications, they are unstable against GAGase-mediated degradation [10].

Tannic acid (TA), a tea polyphenol component with abundant hydroxyl groups, is a strong natural inhibitor of collagenase and hyaluronidase. According to reports, TA could bind to HA via hydrogen bonds, protecting it from enzymatic degradation [11]. We recently found that tea polyphenol could efficiently crosslink acellular ECM, and meanwhile armored copper ions [12], which are often used to fabricate endothelium-mimicking coating [13]. Besides independently exerting beneficial effects in the cardiovascular system, copper also catalyzes endogenous donors to produce nitric oxide (NO) *in vivo*. As an important signaling molecule produced by healthy endothelial cells, NO could accelerate endothelialization, while suppressing platelet aggregation and activation.

Silk fibroin, a natural silkworm-produced fibrous polymer, is a US Food and Drug Administration-approved biomaterial for clinical use. It mainly comprises glycine, alanine, and serine. These amino acid residues endow it with hydrophilicity and outstanding self-cleaning performance, as well as antibacterial and antiadhesion properties [14, 15]. Given that it could reduce thrombosis formation [16] and support endothelial cell growth and colonization [17], silk fibroin is particularly promising for a wide range of cardiovascular applications.

The swim bladder, which allows fish to float freely in water, is rich in collagen, elastin, and GAG. Owing to its abundance and superior mechanical attributes, the swim bladder has various potential applications in tissue engineering [18], especially in cardiovascular contexts [19–21]. Compared to traditional bovine pericardium biomaterial, the swim bladder offers several advantages, including no risk of zoonotic virus transmission and religious restrictions, low antigenicity, superior calcification resistance, and desirable hemocompatibility [22].

**Figure 1:**
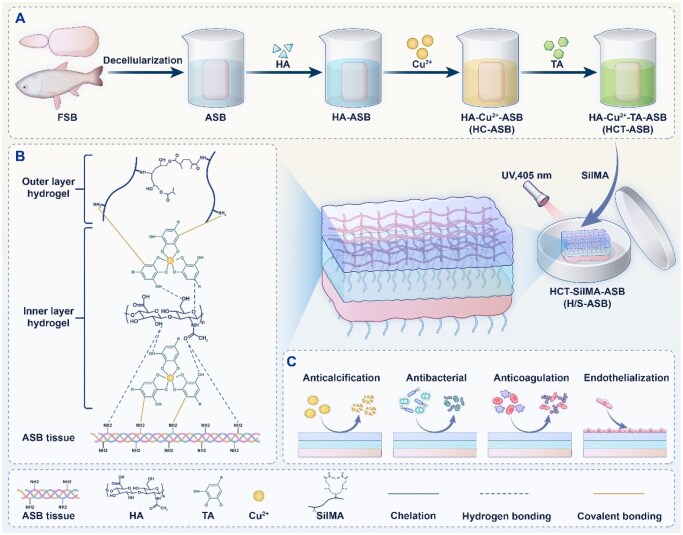
Schematic illustration of (**A**) the preparation, (**B**) formation mechanism, and (**C**) effects of the bilayer hydrogel coating on the ASBs.

Herein, we designed a biomimetic bilayer hydrogel coating that is resistant to thrombosis and calcification using acellular swim bladders (ASBs) as the tissue engineering scaffold (Figure 1). First, an endothelium-mimicking inner hydrogel was synthesized via self-assembly coordination of HA, copper, and TA. A hydrophilic methacrylated silk fibroin (SilMA) was then fabricated as the outer hydrogel coating using light-induced polymerization. The resulting bilayer hydrogel coating was expected to create a thrombosis-resistant antifouling endothelium-mimicking surface, attributable to the integration of NO-generating species (copper–TA coordination complex), glycocalyx macromolecule (HA) and hydrophilic protein (silk fibroin). The bilayer hydrogel-coated ASB scaffolds could also outperform Glut-crosslinked ASB scaffolds in terms of anticalcification. Moreover, besides its antithrombotic and anticalcification properties, this bilayer hydrogel coating could enhance the biostability and biomechanics of ASB scaffolds through an interpenetrating hydrogel network and light crosslinking, and offer antibacterial activity owing to the intrinsic bactericidal properties of TA, copper, and silk fibroin. Overall, our biomimetic bilayer hydrogel coating could functionalize and crosslink tissues, endowing cardiovascular tissue engineering scaffolds with robust antithrombotic and anticalcification properties.

## Materials and methods

### Fabrication of swim bladder scaffolds

Fresh swim bladders (FSBs) of carp were purchased from the local aquaculture market (Shanghai, China) and transported in ice to the laboratory. They were decellularized as described previously [23]. The resultant ASBs were lyophilized and soaked in 2% (w/v) HA (Macklin, China) to fabricate HA-ASBs. They were subsequently immersed in 1.0 µg/mL copper sulfate (CuSO_4_, Macklin, China) solution and treated with 0.15% (w/v) TA solution to obtain HCT-ASBs. Finally, the HCT-ASBs were coated with photocrosslinked SilMA hydrogel following the manufacturer’s instructions (EFL-Tech, China). The resultant ASBs were named as H/S-ASBs, and the armoring of HA within ASB matrix was evaluated through fluorescein isothiocyanate (FITC)-conjugated HA (FITC-HA, Sigma, China), with the results compared with Glut-crosslinked ASBs (Glut-ASBs).

### Residual DNA assay

To visualize DNA material, the FSBs and ASBs were frozen, cut into 5-µm-thick sections, and stained with 4′,6-diamidino-2-phenylindole (DAPI, BEYOTIME, China). Additionally, total DNA was extracted using DNeasy Blood & Tissue kit following the manufacturer’s protocol (Qiagen, The Netherlands). Tissue DNA content was calculated with reference to a DNA concentration and sample weight (ng DNA/mg tissue).

### ECM quantification

Lyophilized samples were weighed, dried under nitrogen gas, and ground into powder. Total GAG content per dry weight was quantified using a colorimetric assay kit (Genmed Scientifics Inc., USA). The absorbance was recorded at 656 nm, and quantification was determined against a standard curve. Collagen and elastin were quantitatively analyzed using an enzyme-linked immunosorbent assay (ELISA) method (Kenuodi, China).

### Surface characterization

The surface morphology and roughness of the coating were characterized using a scanning electron microscope (SEM, Zeiss, Germany). To analyze the chemical composition, an energy dispersive X-ray spectroscopy (EDS) equipped with SEM was employed. The chemical structures were analyzed using attenuated total reflection infrared spectroscopy (ATR-FTIR, Nicolet 6700, Thermo Fisher Scientific). The hydrophilicity alteration of the coating was measured by the water contact angle (WCA, HARKE-SPCAX1, China). The anti-protein adsorption performance of the coating was analyzed using FITC-labeled bovine serum albumin (FITC-BSA) as the model protein.

### Crosslinking degree, biostability, and biomechanics

A ninhydrin assay was performed to measure the degree of crosslinking using a commercially available kit (Zike Biotechnology, China). Tensile testing was performed using a Zwick tensile tester (Zwick GmbH & Co. KG). Biostability was analyzed using collagenase and GAGase degradation assays.

### Antibacterial activity assay

The antibacterial activity of ASB samples was evaluated using *Staphylococcus aureus* (*S. aureus*, Gram-positive bacteria) and *Escherichia coli* (*E. coli*, Gram-negative bacteria) as the model bacteria. First, the tested ASB samples were co-cultured with the model bacteria for 6 h. Next, the surviving bacteria were diluted to a certain concentration, and 100 μL of the diluted bacterial suspension was uniformly spread on a nutrient agar plate. Following incubation at 37°C for 24 h, the number of colonies on the agar plate was photographed and counted [24, 25]. In addition, the resuspended bacterial suspension was incubated in a shaker for 8 h. Bacteria cultured without ASB were set as a control. The absorbance was measured using a microplate reader (SpectraMaxM2e, Molecular Devices, USA) at 600 nm, and the antibacterial rate was calculated. Biofilm inhibition assay was performed in a 24-well plate according to the method described previously [26, 27]. The formed biofilm was stained with crystal violet and photographed. Subsequently, the biofilm was dissolved in 95% ethanol, the absorbance at 570 nm was measured using the microplate reader, and the biofilm inhibition rate was calculated.

### Cell viability assay

An extract test was conducted for cytotoxicity evaluation according to ISO 10993-5: 2009 [28]. Briefly, the ASB samples were sterilized by ultraviolet (UV) light and then immersed in the culture medium at 37°C for 24 h to prepare the ASB extract medium. Next, human umbilical vein endothelial cells (HUVECs) or L929 cells (1 × 10^4^ cells per well) were cultured in the ASB extract medium. The cells were stained with Calcein-AM and propidium iodide to evaluate cell morphology and viability using a live/dead fluorescence staining kit (EFL-Tech, China). Cell metabolic activity was quantitatively analyzed by Cell Counting Kit-8 (CCK-8, Dojindo, Japan).

### Blood experiments

All blood experiments were approved by the Ethics Review Committee for Animal Experimentation of Changhai Hospital. Whole blood samples were collected from healthy New Zealand white rabbits for blood experiments. Hemocompatibility was evaluated using the hemolysis assay. Platelet adhesion on the coating surface was observed using SEM. The concentration of lactate dehydrogenase (LDH) in adhered platelets was assessed using an ELISA kit (YOBIBIO, China). The level of complement 3 (C3) activation was quantified using the C3a ELISA kit (YOBIBIO, China). *In vitro* closed-loop whole blood circulation model was established following previous studies [29, 30]. After 1 h of circulation, the samples were collected and washed with phosphate-buffered saline, then cut into pieces for SEM analysis.

### Animal experiments

All the animal experiments were approved and conducted following the guidance of the Ethics Review Committee for Animal Experimentation of Changhai Hospital (CHEC(A.E)2025-031), which met the ethical requirements of NIH (National Research Council) Guide for the Care and Use of Laboratory Animals. To evaluate the *in vivo* responses including biostability, cell ingrowth, and calcification, the tested samples were implanted subcutaneously into Sprague–Dawley (SD) rats (150–200 g). The *ex vivo* arterio-venous shunt (AV shunt) assay was performed in New Zealand white rabbits (3.0–3.5 kg) to assess the *in vivo* antithrombotic effects of hydrogel-coated ASBs [31, 32]. An *in situ* vascular implantation model was established to assess *in vivo* durability. Briefly, the tested samples were fabricated into small-diameter tubular grafts by rolling them up. The constructed grafts were implanted *in vivo* to repair a segmental defect in rat carotid arteries following a cuff technique protocol [33, 34].

### Histology and immunohistochemistry

Formalin-fixed paraffin-embedded tissues were sectioned, deparaffinized, and stained with hematoxylin and eosin (HE) for morphological examination. Collagen and elastin were stained using Verhoeff van Gieson (EVG) method, while the alizarin red S staining was employed to detect calcification. Immunohistochemistry was performed using a streptavidin–peroxidase complex method (SP kit, Fujian Maixin, China). Primary antibodies included CD34 (Abcam, Cambridge, MA, dilution, 1:150) and α-smooth muscle actin (α-SMA) (Abcam, Cambridge, MA, dilution, 1:200).

### Statistical analysis

All experiments involved in this study were conducted with at least three biological replicates, and the results are expressed as mean ± standard error. The differences between the two groups were evaluated by means of *t*-test or Mann–Whitney non-parametric test. Comparison of more than two groups was performed using one-way analysis of variance followed by Tukey’s multiple comparisons. Statistical analyses were performed using GraphPad Prism 6.0 (GraphPad Software, USA). Statistical significance levels are expressed as *P < 0.05, **P < 0.01, ***P < 0.001, and ****P < 0.0001. Error bars were defined as standard error.

## Results and discussion

### Preparation and characterization of H/S-ASBs

**Figure 2:**
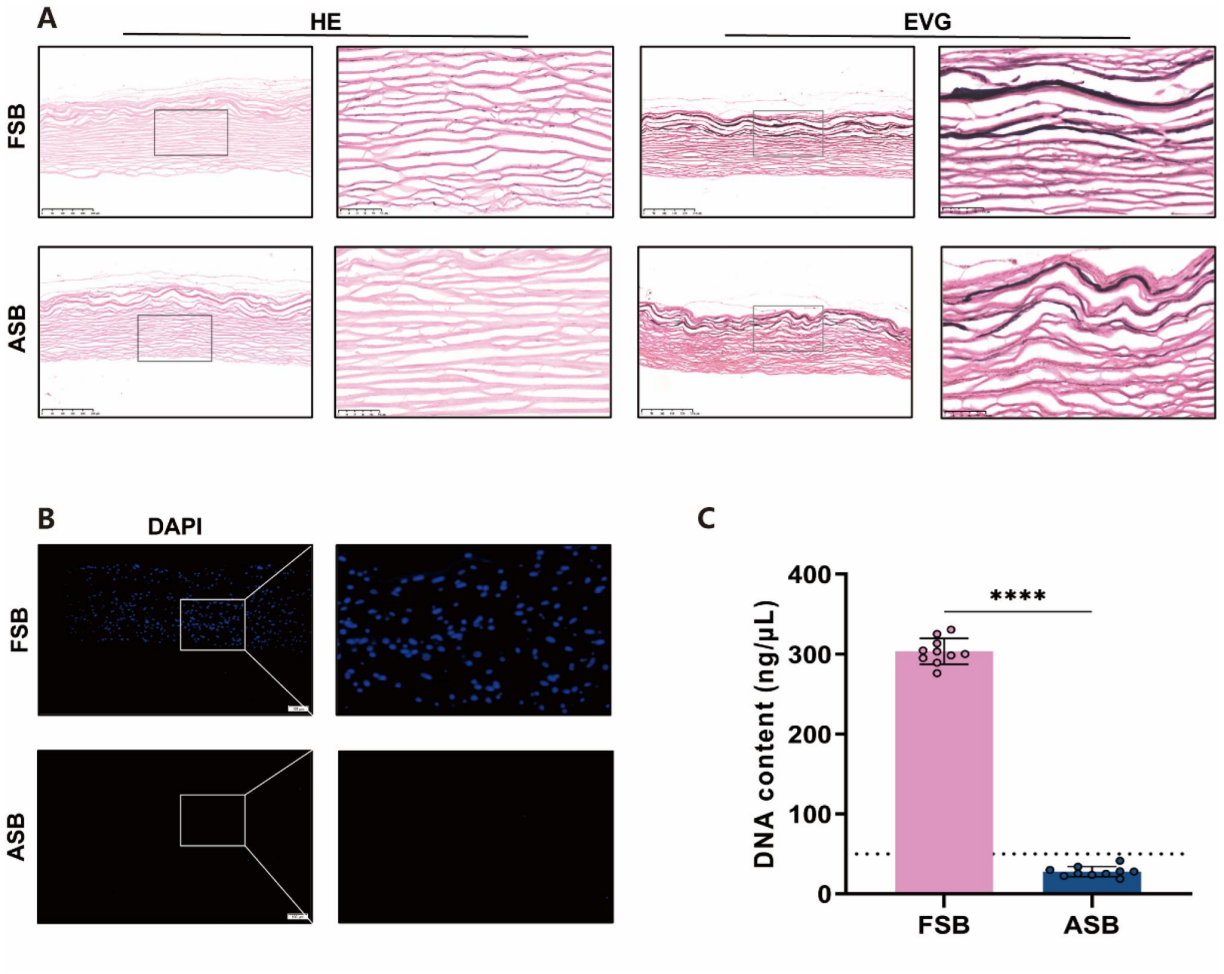
Characterization of ASBs. (**A**) HE and EVG staining. The boxed region is magnified in right panel. Scale bar = 250 µm. (**B**) DAPI staining. The boxed region is magnified in the right panel. Scale bar = 100 µm. (**C**) DNA quantification analysis (*n* = 10). **** *P* < 0.0001.

The swim bladder has been used to construct bioprosthetic heart valves and vascular patches [35]. It is expected to be a candidate material for cardiovascular tissue engineering applications [36]. In this regard, it is noteworthy that cardiovascular grafts fabricated using ASBs exhibited proper mechanical strength and stability, high patency, and low calcification [37]. Recently, Jiang *et al*. prepared an ASB valve device for transcatheter aortic valve replacement, demonstrating fatigue resistance and *in vitro* stability, as well as long-term functionality and durability after implantation *in vivo* [21]. In this study, FSBs were decellularized and used as tissue engineering scaffolds. The prepared ASBs were evaluated by histological analysis. HE (Figure 2A) and DAPI (Figure 2B) staining results revealed that no obvious cellular nucleus was found in ASBs. DNA quantification analysis confirmed that the DNA content of ASBs was below 50 ng DNA/mg dry tissue weight (Figure 2C), indicating the success of decellularization. EVG staining (Figure 2A) showed that collagen and elastin were well preserved. Quantification analysis revealed that the collagen, elastin, and GAG contents did not change much after decellularization (Supplementary Figure S1).

**Figure 3:**
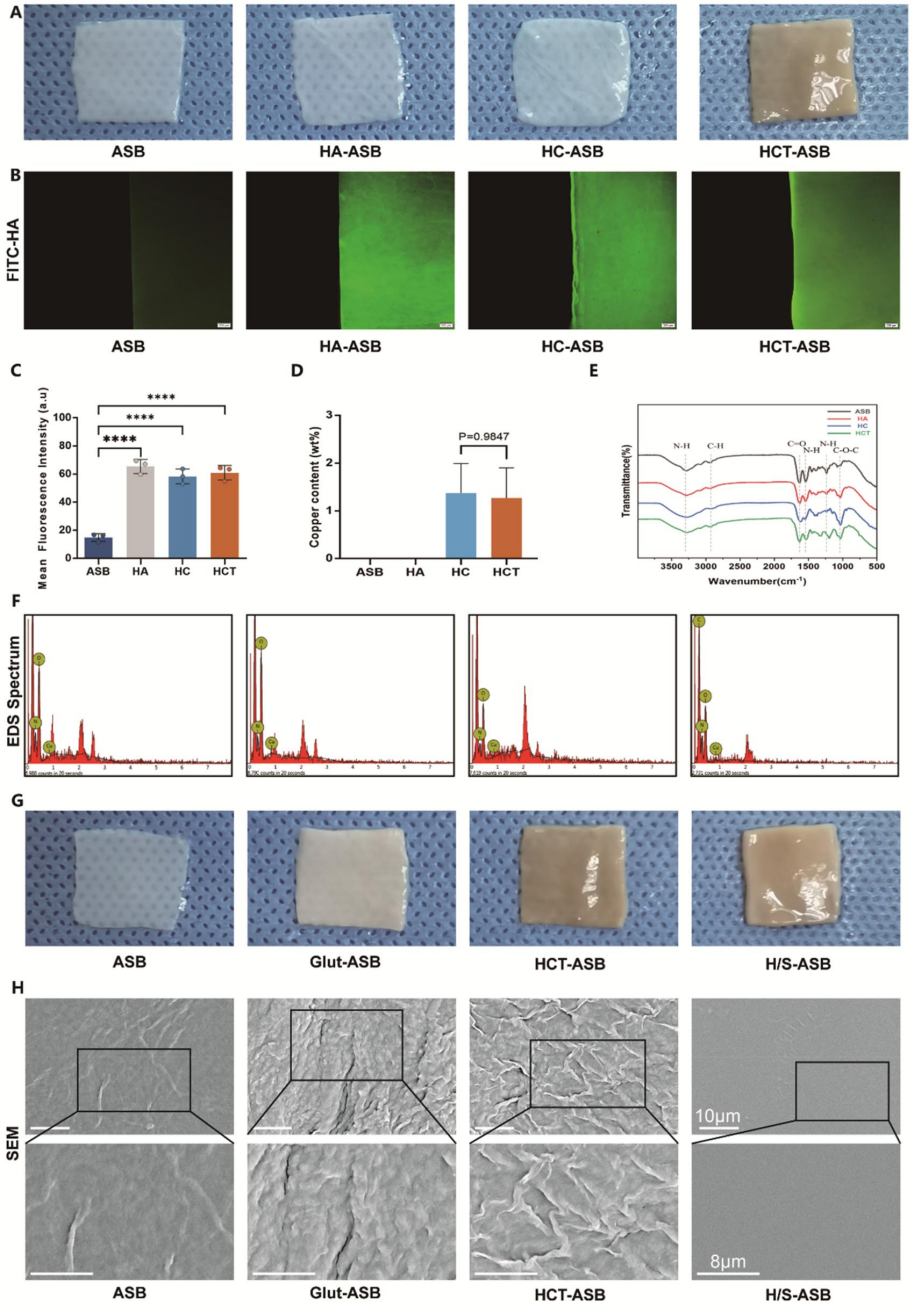
Characterization of H/S-ASBs. (**A**) Representative gross images of ASB, HA-ASB, HC-ASB, and HCT-ASB. (**B**) Fluorescent images of FITC-HA armoring. Scale bar = 200 µm. (**C**) Fluorescent quantification of FITC-HA (*n* = 3). (**D**) Copper content from EDS mapping (*n* = 4). (**E**) Representative FTIR spectra. (**F**) Representative images of EDS spectra. (**G**) Representative gross images of ASB, Glut-ASB, HCT-ASB, and H/S-ASB. (**H**) Microstructure observation from SEM analysis. The boxed region is magnified in the bottom panel. Scale bar = 10 µm and 8 µm. **** *P* < 0.0001.

We also fabricated an endothelium-mimicking inner hydrogel using a simple and cost-effective self-assembly method (Figure 1). Since negatively charged HA could interact with bivalent metal cations [38], ASBs in the dry state were first immersed in HA solution (HA-ASBs) and then armored with copper ions to generate HC-ASBs. Furthermore, given that TA is rich in catechol and pyrogallol groups, it exhibits a strong affinity for various organic and inorganic surfaces [39]. In this regard, it is noteworthy that the side chains of ECM molecules contain numerous amino functional groups, and the carboxyl functional groups in HA side chains provide the sites for interaction with TA [40]. The phenolic hydroxyls in TA could also provide chelation sites for copper ions, forming stable supramolecular metal–phenolic networks. Based on these insights, TA was used to act as a crosslinker to armor HA and copper within the ASB matrix via crosslinking bridges among ASB, HA, copper, and TA, yielding an interpenetrating hydrogel network (HCT hydrogel). Figure 3A shows representative images of ASB scaffolds, and the generated ASBs were denoted as HCT-ASBs.

To visualize HA interpenetration within the ASB matrix, FITC-HA was employed, and fluorescent intensity was measured using a fluorescent spectrophotometer. Compared to ASBs, HA-ASBs, HC-ASBs, and HCT-ASBs showed higher fluorescence intensities (Figure 3B and C). Furthermore, FITC-conjugated HA was evenly distributed across each ASB layer. As a kind of high molecular weight GAG, HA contributes to maintaining ECM integrity [41]. Therefore, we measured total GAG to quantify the amount of HA immobilized in ASBs, and detected an increment of ≈1 µg mg^−1^ GAG in ASBs with exogenous HA supplementation. Elemental analysis with EDS spectra further revealed the presence of copper elements in HC-ASBs and HCT-ASBs (Figure 3D and F). Figure 3E shows the FTIR spectra of ASBs, HA-ASBs, HC-ASBs, and HCT-ASBs. It has been reported that the characteristic peaks of HA were observed at 3406.48, 1617.72, and 1044.11 cm^−1^, which corresponded to the O–H stretching, N–H bending, and C–O–C stretching, respectively [42]. The characteristic peak O–H stretching in the spectra of HA-ASBs, HC-ASBs, and HCT-ASBs became broad, and C–O–C stretching at 1033 cm^−1^ was clearly observed [43], suggesting the interaction between HA and ASB matrix. Compared to HA-ASBs, the amide II in the spectra of HC-ASBs demonstrated a lower wavenumber, a phenomenon attributable to the formation of new chelate bonds between metal ions and C=O bonds weakening C=O bonds in the peptide chain [44]. In comparison, the amide II in the spectra of HCT-ASBs exhibited an opposite shift, influenced by the hydrogen binding of TA to ASBs.

We then fabricated the antifouling outer hydrogel through photopolymerization (Figure 1). As aforementioned, TA has a strong binding affinity to various surfaces due to its strong covalent and noncovalent interactions with almost all material types. Furthermore, TA could form hydrogen bonds with silk fibroin [45], suggesting that HCT-ASBs might conjugate with SilMA through surface-anchored TA. Therefore, to produce SilMA hydrogel coating, HCT-ASBs were covered with SilMA solution in the presence of photoinitiator lithium phenyl(2,4,6-trimethylbenzoyl) phosphinate (LAP), and then irradiated with UV light. The obtained bilayer hydrogel-coated ASBs were denoted as H/S-ASBs (Figure 3G). Additionally, Glut was selected as a highly effective crosslinker for ASBs (Glut-ASBs). According to the SEM results, the matrix structures of Glut-ASBs, HCT-ASBs, and H/S-ASBs became denser than those of ASBs due to the crosslinking (Figure 3H). Particularly, compared to HCT-ASBs, H/S-ASBs exhibited a smooth and confluent surface, indicating that SilMA hydrogel coating improved the smooth appearance of ASB scaffolds. The interface structure between the ASB scaffold and the bilayer hydrogel was presented in Supplementary Figure S2. HCT hydrogel penetrated into the ASB matrix, which made the structure of HCT-ASBs denser than that of ASBs. SilMA hydrogel possessed a porous network structure and was firmly bound to HCT-ASBs without separation at the interfaces, which may be due to the conjugation of TA with SilMA.

### Crosslinking degree, biostability, biomechanics, and antibacterial activity of H/S-ASBs

**Figure 4:**
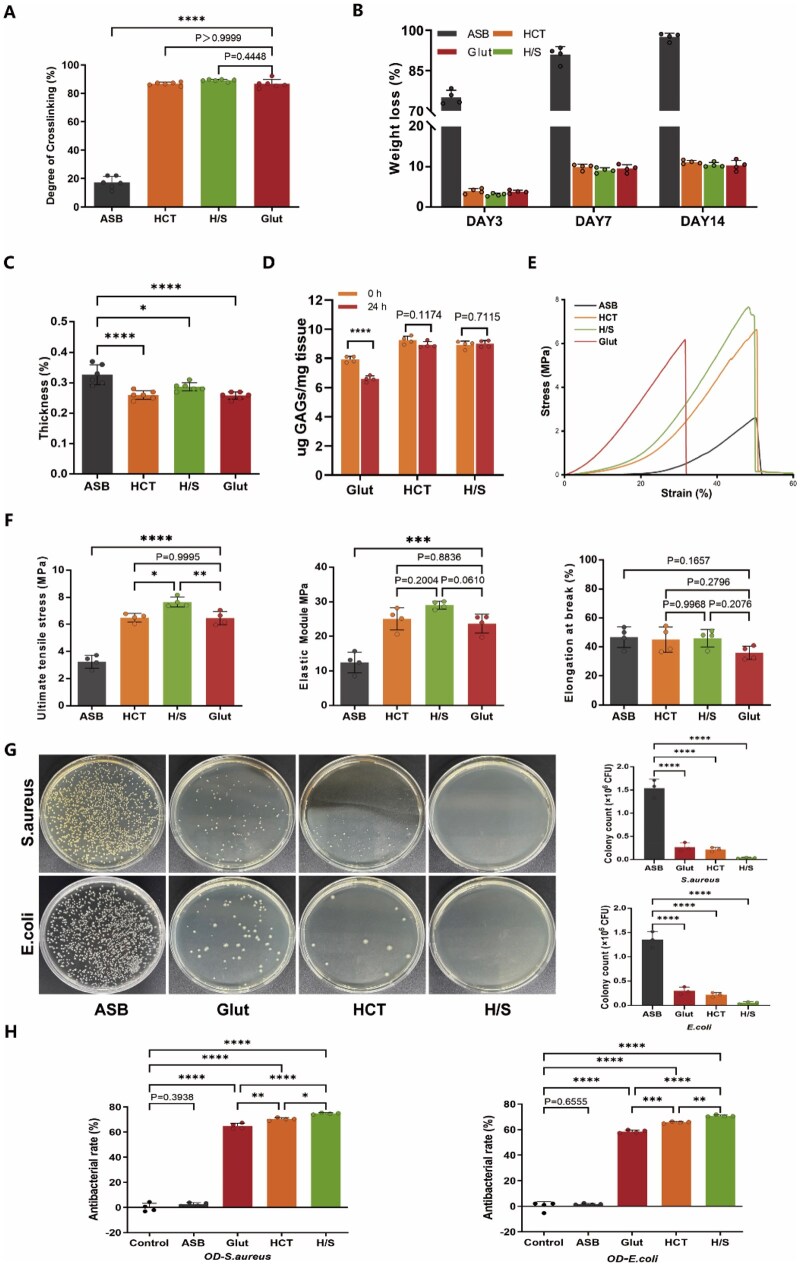
Crosslinking characterization and antibacterial activity of H/S-ASBs. (**A**) The quantitative results of crosslinking degree determined by ninhydrin assay (*n* = 6), (**B**) weight loss after collagenase treatment (*n* = 4), (**C**) tissue thickness (*n* = 6), (**D**) GAG content before and after GAGase treatment (*n* = 4). (**E**) Representative tensile stress–strain curves. (**F**) Quantitation of uniaxial tensile stress, elastic modulus, and elongation at break (*n* = 4). (**G**) Representative images (left) and quantification (right, *n* = 3) of bacterial colonies on the agar plates. (**H**) The antibacterial rates of biopatches against *S. aureus* and *E. coli* (*n* = 4). * *P* < 0.05, ** *P* < 0.01, *** *P* < 0.001 and **** *P* < 0.0001.

Herein, the interpenetrating inner hydrogel network increased the crosslinking degree of ASBs, which, in turn, increased their biostability and mechanical strength. The crosslinking degrees of HCT-ASBs and H/S-ASBs were 86.79 ± 1.15% and 89.16 ± 0.77%, respectively (Figure 4A), with no significant difference from Glut-ASBs (86.78 ± 3.00%). Furthermore, during crimping and deployment, coatings that are too thick are unsuitable for bioprostheses fabrication. Herein, the thickness of Glut-ASBs, HCT-ASBs, and H/S-ASBs was 0.26 ± 0.01 mm, 0.29 ± 0.01 mm, and 0.26 ± 0.01 mm, respectively (Figure 4C), significantly thinner than ASBs (0.33 ± 0.03 mm). These findings indicate that crosslinking made the ASBs denser, and hydrogel coating did not increase thickness.

Notably, ASBs were susceptible to type I collagenase, exhibiting >75% weight loss at day 3 and complete degradation at day 14 (Figure 4B). Conversely, crosslinking prevented rapid degradation of ASBs, with the three crosslinked ASBs (Glut-ASBs, HCT-ASBs, and H/S-ASBs) showing <20% weight loss at day 14. Furthermore, Glut-ASBs, HCT-ASBs, and H/S-ASBs showed no significant difference in terms of collagen stability against type I collagenase.

In previous reports, GAGase easily degraded HA *in vivo* [10]. Despite being an excellent crosslinking agent for collagen, Glut does not stabilize GAG [46–48]. Consistent with previous studies, we found that the GAG content of Glut-ASBs decreased significantly following digestion with hyaluronidase for 24 h (Figure 4D). However, HCT-ASBs and H/S-ASBs showed no significant differences in GAG content both before and after hyaluronidase treatment, implying that TA crosslinking could stabilize HA against hyaluronidase degradation (Figure 4D).

Glut crosslinking is commonly used to strengthen the biomechanical properties of biomaterials. Herein, tensile testing revealed no significant differences in ultimate tensile strength, elastic modulus, and elongation at break between HCT-ASBs and Glut-ASBs (Figure 4E and F), suggesting that the strong intermolecular interactions between TA, copper, HA, and collagen molecules significantly improved ASB biomechanics. Silk possesses high mechanical strength and favorable elasticity; hence, it has long been used in biomedical applications [49]. According to reports, SilMA, a novel silk-modified biomaterial fabricated by adding methacrylate groups to amine residues of silk fibroin, can be rapidly photocured into hydrogel formation while retaining strong mechanical properties [50]. Herein, compared to HCT-ASBs, H/S-ASBs showed a significantly higher ultimate tensile strength. Additionally, H/S-ASBs demonstrated a higher ultimate tensile strength than Glut-ASBs.

Despite significant efforts, implantable devices still get infected with microorganisms during surgery or after surgery, causing bacterial illnesses, such as endocarditis [24, 51]. Some bacteria, such as coagulase-positive *S. aureus*, could also contribute to thrombi formation [52, 53]. Therefore, to create an infection-free environment for cardiovascular applications, we evaluated antibacterial performance against two representative bacterial strains: *S. aureus* and *E. coli*. The ASB group exhibited numerous newly grown colonies, whereas Glut-ASBs and HCT-ASBs markedly reduced the colony formation abilities of the tested microorganisms (Figure 4G), with almost no colonies observed on agar plates for the H/S-ASB group. Quantitative analysis of colony-forming unit revealed that Glut-ASBs, HCT-ASBs, and H/S-ASBs exhibited a higher killing efficiency than that of ASBs (Figure 4G). Next, the antibacterial rate was evaluated using the 600-nm absorbance assay. The results showed that Glut-ASBs, HCT-ASBs, and H/S-ASBs exhibited a significant killing efficiency against both *S. aureus* and *E. coli*, among which, H/S-ASBs displayed the strongest antibacterial activity (Figure 4H).

Biofilms, a layer of bacterial cells embedded in self-produced extracellular polymeric substances, contribute to bacterial resistance and exacerbate infections [54]. The antibacterial efficacy was further validated by assessing the inhibitory effect of the tested samples on biofilm formation. Supplementary Figure S3A demonstrates that crystal violet could stain biofilms from both *S. aureus* and *E. coli*, resulting in a dark purple appearance. Almost no biofilm was formed in the groups of Glut-ASBs, HCT-ASBs, and H/S-ASBs, whereas a large number of intact biofilms were formed in the groups of blank control and ASB groups. Meanwhile, the 570-nm absorbance assay indicated that the H/S-ASBs have an outstanding biofilm inhibition effect, which was consistent with the abovementioned results (Supplementary Figure S3B).

### Biocompatibility and anticalcification of H/S-ASBs

**Figure 5:**
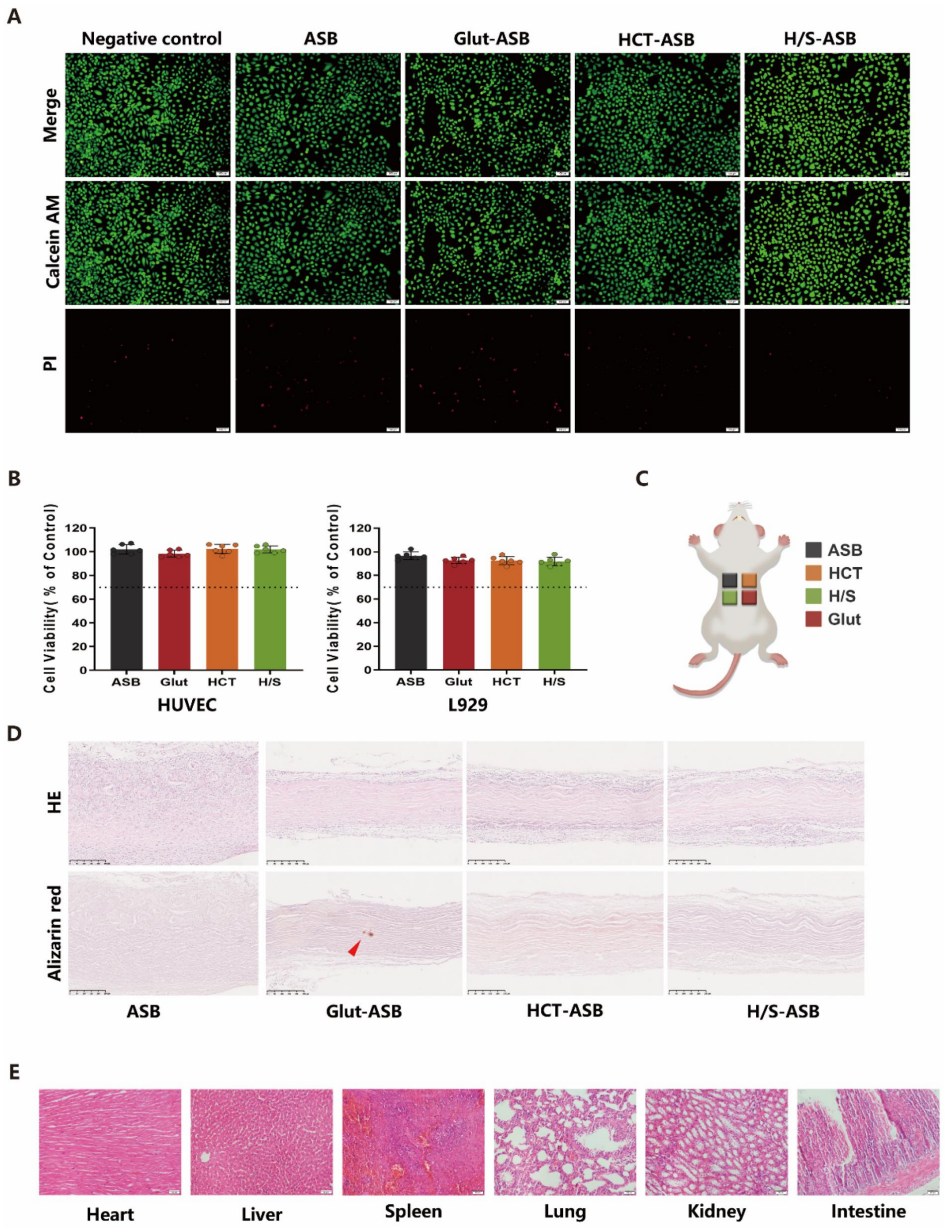
Biocompatibility and anticalcification of H/S-ASBs. (**A**) Cell viability of HUVECs was assessed using live/dead assay. Scale bar = 100 µm. (**B**) Cell metabolic activity of HUVECs and L929 cells determined by CCK-8 assay (*n* = 6). (**C**) Schematic illustration showing *in vivo* rat subcutaneous implant model. (**D**) HE and Alizarin red staining of ASB explants. Scale bar = 250 µm. Red triangle, calcification. (**E**) HE staining of major organs from rat implant model. Scale bar = 50 µm.

Implant biomaterials should be cytocompatible before clinical application. Herein, the fabricated ASBs were subjected to cytotoxicity evaluation using two assays. Cell morphology and viability were analyzed via live/dead fluorescence staining, while cell metabolic activity was quantitatively examined using the CCK-8 assay. The cells cultured in standard culture medium served as the negative control group. Supplementary Figure S4 shows the optical images of HUVECs and L929 fibroblasts after incubation with the different extract media for 3 days. As in the negative control group, HUVECs incubated with extract media of ASBs, Glut-ASBs, HCT-ASBs, and H/S-ASBs showed an irregular circular shape and clear cellular outlines. Similarly, L929 fibroblasts incubated with different extract media also showed regular-shaped morphologies with intact outlines. The live/dead assay confirmed the above phenomenon (Figure 5A and Supplementary Figure S5). Moreover, HUVECs and L929 fibroblasts in the sample extract media showed high viability, as evidenced by the almost total green staining relative to the negative control. To quantitatively assess the cytotoxicity of the fabricated ASBs, each group’s cell metabolic activity was analyzed using the CCK-8 assay. The metabolic activity of HUVECs and L929 fibroblasts exceeded 90% under treatment with extract media in different groups (Figure 5B). Notably, the ISO 10993-5: 2009 guidelines [28] define a cytotoxic effect as >30% reduction in cell metabolic activity compared to the negative control. Therefore, the CCK-8 results confirmed the favorable cytocompatibility of the fabricated ASB scaffolds.

To evaluate the *in vivo* host response, the tested samples were implanted subcutaneously into SD rats (Figure 5C) for 30 days. According to the HE staining results, the ASB explants were almost degraded and completely infiltrated by host cells (Figure 5D). Furthermore, a number of fibroblast-like cells infiltrated the surrounding acellular regions of both HCT-ASB and H/S-ASB explants, whereas the cells could not infiltrate Glut-ASB explants. Alizarin red staining further revealed that Glut-ASB explants exhibited focal calcium deposition, with no visible calcification observed in the explants of the ASB, HCT-ASB, and H/S-ASB groups, confirming that Glut crosslinking accelerated the calcification process. We further evaluated the *in vivo* toxicity of the tested scaffolds; all rats showed no adverse reactions. Moreover, HE staining of the major organs demonstrated that the implanted scaffolds caused no severe inflammatory responses or necrosis, with clear microstructures of the heart, liver, spleen, lung, kidney, and intestine, and regularly arranged cellular morphogenesis (Figure 5E).

### *In vitro* hemocompatibility and anticoagulation capability of H/S-ASBs

**Figure 6:**
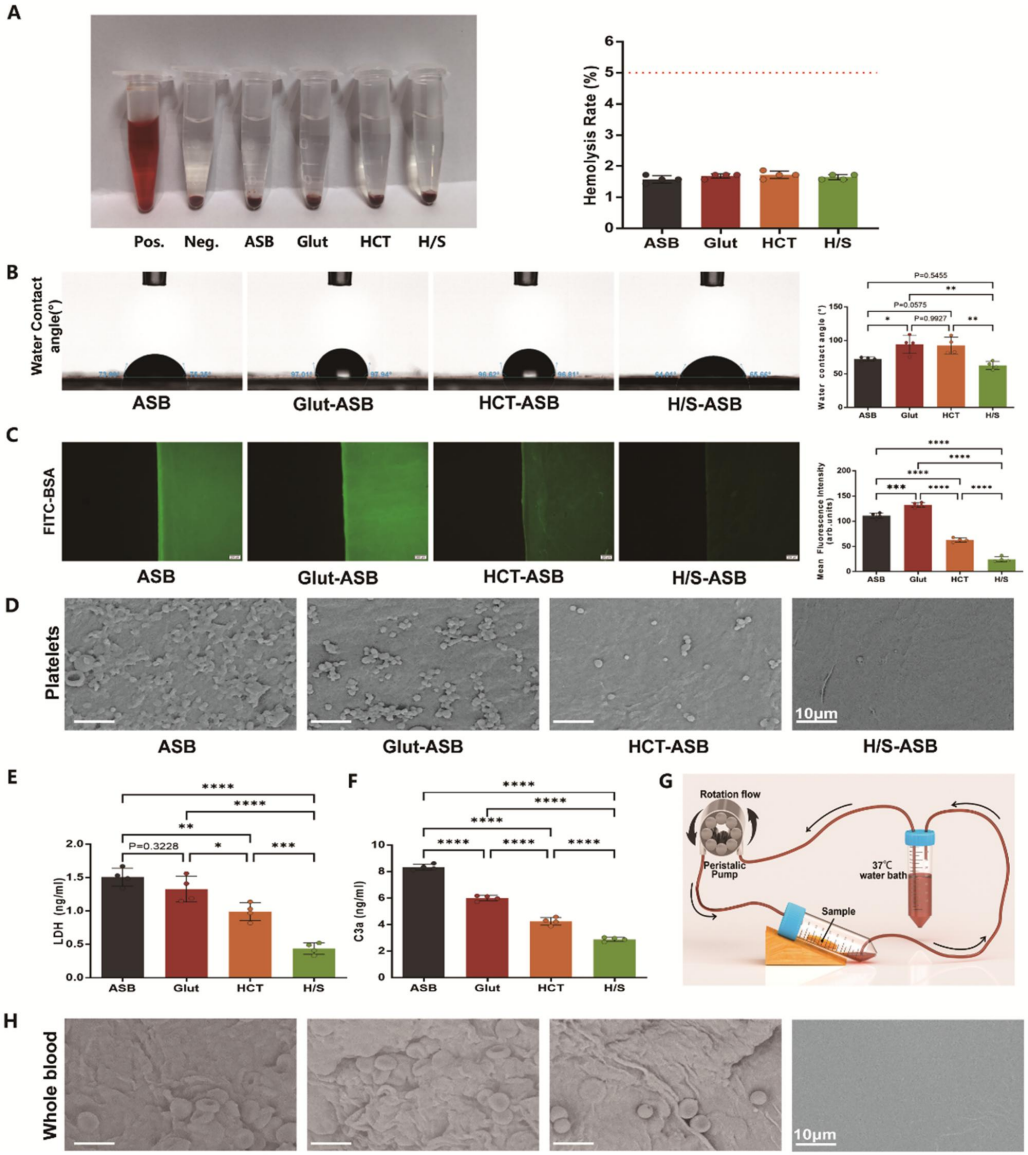
Hemocompatibility of H/S-ASBs. Representative images and quantitative results of (**A**) hemolysis effect (*n* = 4), (**B**) WCA (*n* = 4), and (**C**) FITC-BSA absorption (*n* = 4). Scale bar = 200 µm for **C**. (**D**) SEM images of ASB scaffolds after treatment with PRP. Scale bar = 10 µm. The quantitative result of (**E**) platelet adhesion determined by LDH in PRP (*n* = 4), and (**F**) C3a in the supernatant detected using ELISA assay (*n* = 4). (**G**) Schematic illustration for *in vitro* closed-loop whole blood circulation model. (**H**) SEM images of ASB scaffolds after treatment with whole blood. Scale bar = 10 µm. * *P* < 0.05, ** *P* < 0.01, *** *P* < 0.001 and **** *P* < 0.0001.

Blood-contacting biomaterials should be hemocompatible before clinical applicability, and according to the relevant standards for medical devices, the hemolysis rate should be <5% [55]. Compared with the positive control group, all experimental groups experienced no obvious hemolysis incidents (Figure 6A). Furthermore, the hemolysis rate for ASBs, Glut-ASBs, HCT-ASBs, and H/S-ASBs was 1.58 ± 0.12%, 1.69 ± 0.07%, 1.72 ± 0.12%, and 1.65 ± 0.08%, respectively, all meeting hemolysis rate standards.

In clinical applications, thrombosis could limit the durability and safety of blood-contacting biomaterials, a phenomenon attributable to coagulation abnormalities such as non-specific protein adsorption and platelet adhesion. These coagulation events are reported to link to biomaterial wettability as hydrogen bonding with water could inhibit protein and platelet attachment [56]. Consequently, the surfaces of the tested scaffolds were analyzed using the WCA assay (Figure 6B). The WCA of ASBs was 72.32 ± 2.84°, later increasing to 94.33 ± 13.83° after Glut crosslinking, a phenomenon attributable to the fact that Glut crosslinking might have altered the protein structure, reducing the number of hydrophobic amino acids. On the other hand, the WCA of HCT-ASBs was 92.44 ± 12.43°, higher than that of ASBs and comparable to that of Glut-ASBs. However, compared to HCT-ASBs and Glut-ASBs, a significantly lower WCA (62.84 ± 5.89°) was observed after the SilMA hydrogel coating of HCT-ASBs. This improvement could be attributed to the presence of hydrophilic groups including carboxyl groups, hydroxyl groups, and amino groups in silk fibroin [57].

Subsequently, we used FITC-BSA as a model protein to evaluate the grafts’ capability to resist non-specific protein absorption (Figure 6C). The mean fluorescence intensities of ASBs, Glut-ASBs, HCT-ASBs, and H/S-ASBs were 111.00 ± 5.44%, 132.50 ± 4.72%, 62.75 ± 0.12%, and 24.40 ± 5.14%, respectively. These findings indicate that H/S-ASBs exhibited the lowest fluorescence intensity, significantly lower than that of ASBs, Glut-ASBs, and HCT-ASBs. Moreover, HCT-ASBs were more resistant to BSA adsorption compared to ASBs and Glut-ASBs, suggesting that the HCT and SilMA hydrogel coatings formed a bilayer-surface hydration layer that significantly enhanced the anti-protein adhesion ability of ASBs. Additionally, Glut-ASBs adsorbed much more protein than ASBs, likely due to their hydrophobic surface and the Schiff reaction between the residual aldehyde group of Glut and the amino group of protein.

Given that the bilayer hydrogel coating could improve the hydrophilicity and antifouling properties of ASBs, we subsequently performed platelet adhesion and activation experiments. According to SEM analysis (Figure 6D), H/S-ASBs exhibited excellent anticoagulation activity in platelet-rich plasma, with minimal platelet adhesion. Platelet quantitation (Figure 6E) using LDH assay revealed comparable LDH values for ASBs (1.51 ± 0.14 ng mL^−1^) and Glut-ASBs (1.33 ± 0.19 ng mL^−1^). Additionally, compared to ASBs and Glut-ASBs, HCT-ASBs (0.99 ± 0.13 ng mL^−1^) showed a significantly lower LDH value. Furthermore, compared to HCT-ASBs, H/S-ASBs (0.44 ± 0.09 ng mL^−1^) showed a significantly lower LDH value.

The level of C3 activation was indicated by the platelet concentration of C3a, a cleaved component of C3. The specific order of the C3a value was (Figure 6F): ASBs (8.34 ± 0.21 pg mL^−1^) > Glut-ASBs (6.01 ± 0.20 pg mL^−1^) > HCT-ASBs (4.25 ± 0.29 pg mL^−1^) > H/S-ASBs (2.90 ± 0.15 pg mL^−1^), aligning with the LDH results, which demonstrated that H/S-ASBs and HCT-ASBs caused a significantly weaker complement activation compared to ASBs and Glut-ASBs (Figure 6F).

A closed-loop circulation was subsequently established to mimic blood circulation (Figure 6G). After constant whole blood perfusion through scaffold surfaces, more red blood cells (RBCs) were visualized on ASBs, Glut-ASBs, and HCT-ASBs, while H/S-ASBs showed minimal blood cell adhesion (Figure 6H).

These findings collectively suggest that the HCT hydrogel coating improved the antifouling capability of ASBs, a phenomenon that the SilMA outer hydrogel further enhanced.

### *Ex vivo* arteries-veins shunt anticoagulation performance

**Figure 7:**
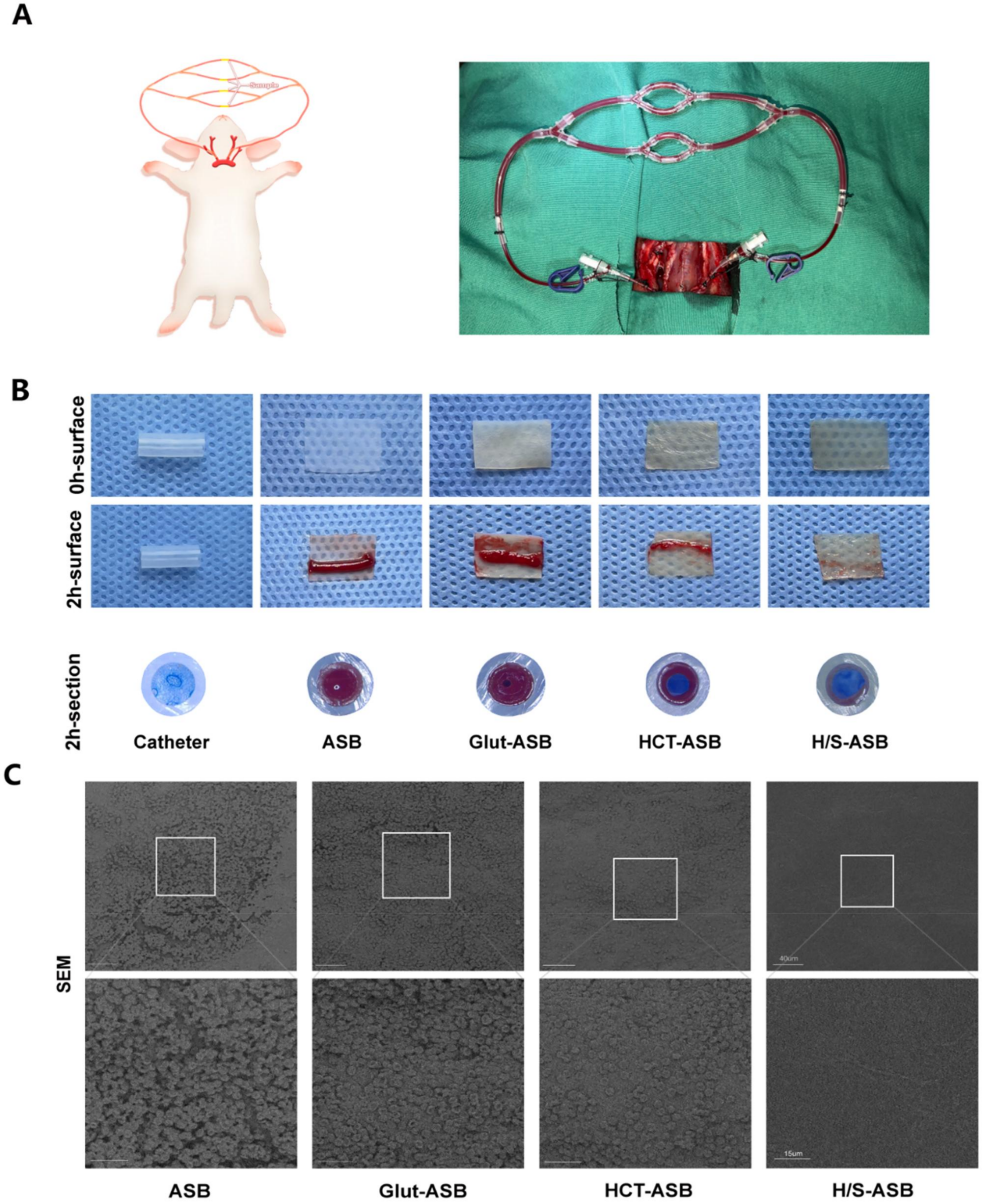
*Ex vivo* anticoagulation performance of H/S-ASBs. (**A**) Illustration for *ex vivo* AV shunt assay. (**B**) Images of ASB scaffolds before and after AV shunt assay. (**C**) SEM images of ASB scaffolds after AV shunt assay. The boxed region is magnified in the bottom panel. Scale bar = 40 and 15 µm.

The *ex vivo* AV shunt assay could realistically simulate the complex hematologic environment that a cardiovascular biomaterial would face post-implantation [58]. In this regard, we performed the *ex vivo* AV shunt assay to evaluate the antithrombogenicity of the tested ASB scaffolds in a rabbit model, in which blood could flow from the carotid artery to the carotid veins and contact the scaffolds at a constant flow rate (Figure 7A). After 2 h of circulation, significant thrombosis formed on the surface of ASBs and Glut-ASBs, with thrombosis also occurring on the HCT-ASB surface, while the H/S-ASB surface remained clean (Figure 7B). From the section, ASBs and Glut-ASBs were completely occluded due to thrombus, while HCT-ASBs still had blood flowing through despite the lumen showing thrombus formation. The four groups were further examined for differences in anticoagulation performance using SEM analysis. The surfaces of ASBs and Glut-ASBs showed the highest amount of coagulation matrix featuring RBCs and fibrin, whereas H/S-ASBs showed no obvious RBCs and fibrin deposition (Figure 7C). Taken together, these findings collectively indicate that the HCT hydrogel coating improved the anticoagulation performance of ASBs, a phenomenon that the SilMA outer hydrogel further enhanced.

### *In situ* vascular replacement performance

**Figure 8:**
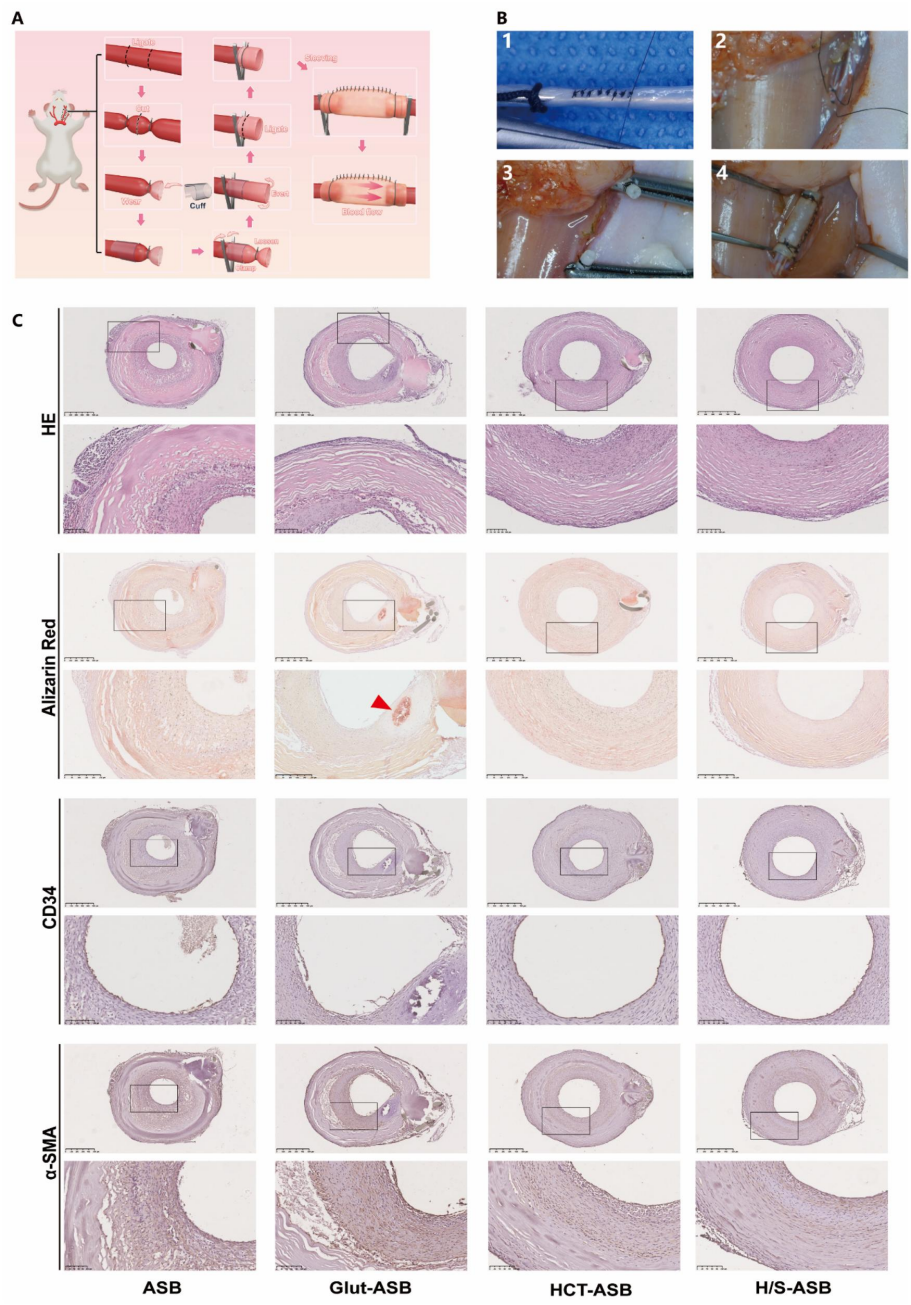
*In situ* vascular replacement performance of H/S-ASBs. (**A**) Illustration for *in situ* vascular replacement. (**B**) Procedure of *in situ* vascular replacement: (1) ASB graft preparation, (2) vascular isolation, (3) vascular transection, and (4) ASB graft suture. (**C**) HE, alizarin red, and immunohistochemical staining of ASB implants. The boxed region is magnified in the bottom panel. Scale bar = 500 and 100 µm. Triangle, calcification.

Having confirmed their desirable biophysical and excellent anticoagulation and anticalcification properties, the prepared ASBs were implanted into the carotid arteries of SD rats to evaluate their practical performance. Figure 8A and B illustrate the vascular replacement procedure. Whereas ASBs were too soft to cut and suture, Glut-ASBs, H/S-ASBs, and HCT-ASBs were easy to cut and suture, suggesting that crosslinking could mechanically enhance ASBs.

After 1 month, the implanted conduit was removed for histological analysis. The circumferential integrity of ASB grafts was damaged, and vascular stenosis occurred due to intimal hyperplasia (Figure 8C). However, the Glut-ASB, H/S-ASB, and HCT-ASB grafts maintained their vascular integrity without structural loosening or delamination, implying that the crosslinked ASBs exhibited long-term structural stability under the hemodynamic arterial microenvironment *in vivo*. Although Glut-ASB grafts preserved their primary structures, they showed poor tissue regeneration, with few cells penetrating the conduit wall. Furthermore, alizarin red S staining demonstrated calcification in Glut-ASB grafts, while no calcification, as anticipated, was detected in H/S-ASB and HCT-ASB grafts.

A fully formed endothelium is resistant to thrombosis, infection, and inflammatory reaction, highlighting the significance of rapid endothelialization to the long-term performance of cardiovascular grafts [59, 60]. After implantation, endothelial cell coverage was superior in HCT-ASB and H/S-ASB grafts compared to Glut-ASB grafts (Figure 8C). Furthermore, both HCT-ASB and H/S-ASB grafts exhibited a contiguous luminal covering of CD34^+^ endothelial cells. Therefore, it is noteworthy that copper and NO release from the HCT hydrogel could enhance the proliferation and migration of endothelial cells, thus accelerating endothelialization.

When tissue engineering scaffolds are implanted as vascular grafts, circumferential orientation remodeling of contractile smooth muscle cells (SMCs) within the grafts plays a key role in providing the structural support required to maintain blood pressure, thus crucially mediating functional blood vessel maturation and reconstruction [61]. As one of the earliest markers of smooth muscle lineage differentiation, α-SMA is the most abundant contractile protein in vascular SMCs [62]. Herein, we characterized SMC infiltration and distribution using α-SMA. Compared to Glut-ASB grafts, α-SMA^+^ SMCs extensively penetrated the interior of HCT-ASB and H/S-ASB grafts. Moreover, there were significantly more α-SMA^+^ SMCs within the interior of H/S-ASB grafts compared to HCT-ASB grafts, suggesting that the SilMA hydrogel further enhanced SMC remodeling. Notably, NO could inhibit SMC proliferation while up-regulating contractile protein expression in SMCs [63]. Specifically, silk fibroin stiffness could enhance the phenotypic maturation of SMCs [64].

## Conclusion

Herein, an endothelium-mimicking hydrophilic bilayer hydrogel coating was designed and fabricated on ASB scaffolds. It comprised two functional layers: (i) an endothelium-mimicking HCT hydrogel layer, and (ii) a hydrophilic and antifouling SilMA hydrogel layer. In addition to enhancing the biostability and biomechanics of ASB scaffolds, this bilayer hydrogel coating also endowed them with multiple functionalities including anticoagulation, antibacterial activity, and anticalcification properties. Furthermore, the *in vivo* study in a rat carotid aorta replacement model further demonstrated its effectiveness in promoting endothelialization, enhancing vascular remodeling, preventing calcification and thrombosis formation, and ultimately improving ASB durability. Therefore, the endothelium-mimicking hydrophilic hydrogel coating holds great promise as a feasible strategy for fabricating cardiovascular tissue engineering scaffolds.

Besides, we summarized the main properties of representative implantable biomaterials (Supplementary Table S1) and hydrogel coating systems (Supplementary Table S2) for cardiovascular applications. Comparative analysis revealed that this bilayer hydrogel-coated ASBs have several limitations that need to be addressed, despite their valuable insights. First, the fabrication strategy for this biomimetic bilayer hydrogel coating requires further optimization to achieve long-term effects that mimic the endothelium. Second, although the H/S coating enhanced SMC remodeling in ASB vascular grafts, this effect as well as other markers and underlying regulatory mechanisms should be explored through *in vitro* and *in vivo* studies. Third, the promising results of the H/S-ASB grafts were only demonstrated in small animal models, necessitating the use of larger animal models and clinically relevant surgical approaches in further studies to evaluate the graft’s antithrombotic and anticalcification properties and advance clinical translation.

## Supplementary Material

rbaf122_Supplementary_Data
