# Supplementary material for: Biomimetic bilayer hydrogel coating with antithrombotic and anticalcification properties for cardiovascular tissue engineering application
Source: Regen Biomater. 2025 Dec 1;13:rbaf122. doi: 10.1093/rb/rbaf122 (PMC12869794; doi:10.1093/rb/rbaf122)
Supplement: rbaf122_Supplementary_Data [file rbaf122_supplementary_data.docx]

**Biomimetic bilayer hydrogel coating with antithrombotic and anticalcification properties for cardiovascular tissue engineering application**

**Supplementary data**

**Materials and methods**

**Decellularization of swim bladder**

Fresh swim bladders (FSBs) of carp were harvested from the local aquaculture market (Shanghai, China) and transported on ice to the laboratory. Decellularization was performed following the protocol as previously described with some modification [1]. Briefly, FSBs were immersed in decellularized solution containing 1% Triton X-100 (Sigma Aldrich, USA) and 0.5% sodium deoxycholate (Sigma Aldrich, USA) for 12 h at 37°C. After rinsing with sterile phosphate buffered saline (PBS) for 3 d, the obtained ASBs were sterilized using 70% alcohol and rinsed in sterile PBS.

**Fabrication of hydrogel coated ASBs**

Acellular swim bladders (ASBs) were lyophilized to dry state using a vacuum freeze dryer (HETO, China). Lyophilized ASBs were soaked in 2% (w/v) hyaluronic acid (HA, Macklin, China, pH = 7.35) solutions at 4°C for 24 h in a shaker (100 rpm) to obtain HA-ASBs. Next, HA-ASBs were immersed in 1.0 µg/mL copper sulfate (CuSO_4_, Macklin, China) solution at 4°C in the shaker (100 rpm). After 24 h, the obtained ASBs (HC-ASBs) were washed with PBS to remove unbounding copper ions. Then, HC-ASBs were treated with 0.15% tannic acid (TA) solution for 24 h at 37°C to obtain HCT-ASBs.

Methacrylated silk fibroin (SilMA) hydrogel coating was performed according to the manufacturer's instructions (EFL-Tech, China). In brief, SilMA solution at the concentration of approximately 10% (w/v) was prepared and the [photoinitiator](https://www.sciencedirect.com/topics/medicine-and-dentistry/photoinitiator) lithium phenyl (2,4,6-trimethylbenzoyl) [phosphinate](https://www.sciencedirect.com/topics/pharmacology-toxicology-and-pharmaceutical-science/phosphinate) (LAP) was added into the mixture at 0.2% [2]. Then, HCT-ASBs were immersed in the prepared SilMA/LAP solution and photocrosslinked by exposure to ultraviolet (UV) light and the obtained ASBs were denoted as H/S-ASBs.

To visualize the armoring of HA within ASB matrix, ASB scaffolds (~1 cm^2^) were placed in a 24-well plate and incubated with 500 μL fluorescein isothiocyanate (FITC)-conjugated HA (FITC-HA, Sigma, China) at 4°C for 24 h. After rinsing with PBS for three times, the scaffolds were observed with fluorescence microscope.

For comparison purposes, ASBs and [Glut](https://www.sciencedirect.com/topics/medicine-and-dentistry/glutaraldehyde) crosslinked ASBs (Glut-ASBs) were prepared. [Glut](https://www.sciencedirect.com/topics/medicine-and-dentistry/glutaraldehyde) crosslinking was performed as the previous study [3]. The ASBs were fixed in 0.6% Glut (Sinopharm Chemical Reagent, China) in 50 mM 4-(2-hydroxyethyl)-1-piperazineethanesulfonic acid (HEPES)-buffered saline (pH 7.4) for 24 h at room temperature. Subsequently, the solution was replaced by 0.2% Glut and stored for 6 d before used.

**4′,6-diamidino-2-phenylindole (DAPI) staining**

To visualize DNA material, FSBs and ASBs were frozen and cut into 5-µm-thick sections. Subsequently, sections were stained with DAPI (BEYOTIME, China) for 1 min. After rinsing with PBS, the slides were evaluated with a fluorescent microscope.

**DNA extraction and quantification**

Total DNA was extracted from FSBs and ASBs using DNeasy Blood & Tissue kit according to the manufacturer's protocol (Qiagen, The Netherlands). DNA concentration was measured using a NanoDrop 2000 (MaestroNano, USA), and tissue DNA content was calculated according to the DNA concentration and sample weight (ng DNA/mg tissue).

**Glycosaminoglycan (GAG) quantification**

Lyophilized samples were weighed and dried under nitrogen gas. Total GAG content per dry weight was determined using a colorimetric assay kit (Genmed Scientifics Inc., USA). A pinkish-red color is indicative of the combination between tissue GAG and 1,9-dimethylmethylene blue. Absorbance was measured at 656 nm, and quantification was determined against a standard curve.

**Collagen and elastin quantification**

Lyophilized samples were weighed and dried under nitrogen gas. Then, 50 mg of the tissue powder was dispersed in 500 μL of PBS at 37°C for 24 h. After centrifugation for 20 min, the supernatant was collected and measured by using an ELISA kit (Kenuodi, China) according to the manufacturer's instructions.

**Scanning electron microscopy (SEM) and corresponding energy dispersive X-ray spectroscopy (EDS)**

The tested samples were cut and fixed in 2.5 % glutaraldehyde solution overnight. After dehydration and drying at room temperature, the surface morphology of the tested samples was observed by Zeiss Ultra 55 SEM (Zeiss, Germany). To analyze chemical composition, an EDS equipped with SEM was used.

**Fourier transform infrared (FTIR) spectroscopy**

The samples were frozen and lyophilized and the acquisition of infrared spectra of all samples were carried out using a FTIR Nicolet 6700 Transmittance spectrometer (Thermo Fisher, USA). Absorbance spectrums were recorded at a resolution of 4 cm^-1^ in the range of 4000 to 400 cm^-1^. Data were analyzed by quantitative peak information.

**Degree of crosslinking**

A ninhydrin assay was performed to measure the amount of free amino groups of each sample using a commercially available kit (Zike Biotechnology, China). The tested samples were lyophilized and heated with a ninhydrin solution for 20 min. The optical absorbance of the solutions was recorded with a spectrophotometer at 570 nm using glycine at various known concentrations as standard. Degree of crosslinking is calculated following the equation:

Degree of crosslinking (%) = (1 – amine content in sample/amine content in non-crosslinked sample) × 100%.

**Tensile testing**

Experiments were carried out using a Zwick tensile tester (Zwick GmbH & Co. KG). The samples were cut into 2 (length) × 1 (width) cm rectangular strips. The mean thickness of each sample was determined by a series of measurements at four different points using a Mitutoyo digital micrometer. Samples were attached to grips. The tensile testing was performed at 5 mm/min until failure. All testing was conducted at room temperature.

**Hydrophilicity**

The hydrophilicity of the sample surface was evaluated by the water contact angle (WCA), which was measured by a contact angle measurement instrument (HARKE-SPCAX1, China) at room temperature. A micropipette was used to dispense 2 µL water droplet on to the surface of the samples. The droplets were then photographed and recorded and the WCA value was calculated by image analysis software.

**Cell culture and ASB extract medium preparation**

L929 fibroblasts were obtained from the American Type Culture Collection (ATCC) and cultured in Dulbecco’s modified Eagle’s medium (DMEM) (Gibco, UK) supplemented with 10% fetal bovine serum (FBS) (Gibco, UK), 100 U/ml penicillin (Gibco, UK) and 100 μg/ml streptomycin (Gibco, UK). Human umbilical vein endothelial cells (HUVECs) were cultured in endothelial cell medium (Gibco, UK) containing 10% FBS, 100 U/ml penicillin and 100 μg/ml streptomycin at 37°C in a humidified atmosphere of 5% CO_2_.

The samples were sterilized by 75% alcohol and then washed with PBS. ASB extract medium preparation was performed with a ratio of surface area to extract medium volume of 3.0 cm^2^/mL, according to ISO 10993-12: 2012 [4]. In brief, the samples were incubated in culture medium supplemented with 10% FBS medium under standard cell culture conditions (95% [relative humidity](https://www.sciencedirect.com/topics/engineering/relative-humidity), 37 °C and 5% CO_2_). After a 24 h incubation, the supernatant was collected as extract medium. Subsequently, extract medium was centrifuged for 5 min at 4°C and 3,000 rpm to remove debris and then stored at −80°C.

**Live/dead fluorescence staining**

The [cell morphology](https://www.sciencedirect.com/topics/engineering/cell-morphology) and viability were qualitatively evaluated by Calcein-AM and propidium iodide (PI) fluorescence staining using the live & dead viability assay (EFL-Tech, China). In brief, HUVECs and L929 cells (1×10^6^ cells per well) were respectively seeded in 12-well culture plates and cultured overnight. Subsequently, the cell medium was replaced by ASB extract medium. After incubation for 72 h, the cell morphologies cultured in the different extract medium were observed and imaged using inverted microscope (Olympus, Japan). Subsequently, extract media were removed, and the cells were gently rinsed with PBS. Afterward, PI staining solution was added to each test well and samples were stained for 10 min in darkness. After rinsing with PBS, Calcein AM staining solution was added to each test well and samples were stained for 20 min in darkness. After rinsing with PBS, the cells were observed and imaged with a fluorescence microscope (Olympus, Japan). Representative areas of the cell layer were photographed to document the amount of living and dead cells, stained by Calcein AM and PI into green and red, respectively.

**Cell metabolic activity assay**

Cell metabolic activity in different groups was quantitatively analyzed using the CCK-8 assay (Dojindo, Japan). Briefly, HUVECs and L929 cells (1×10^4^ cells per well) were respectively seeded in 96-well culture plates and cultured overnight. Subsequently, the cell medium was replaced by 100 µL ASB extract medium. After incubation for 72 h, extract medium were replaced by 100 μL fresh culture medium. Then, 10 μL of CCK-8 reagent was added to each test well and the cultures were incubated for 3 h, following manufacturer’s instructions. The optical density in each well was measured using a microplate reader (SpectraMaxM2e, molecular devices, USA) at 450 nm. The cells incubated with culture medium were used as a negative control, and the pure culture medium was used as a blank control. The cell viability was calculated as follows [5]:

Cell viability (%) = (*OD*_s_ - *OD*_b_) / (*OD*_n_ - *OD*_b_) × 100%

where ODs, ODb, and ODn are the absorbance of the sample, blank control, and negative control, respectively.

**Collagenase degradation assay**

Samples were lyophilized and ground into powders and weighed (initial dry weight). Then, the samples (40 mg) were treated with collagenase type I solution (1.5 mg/mL, Sigma Aldrich, Germany) containing 3.0 µM calcium chloride. At every predetermined time point, the samples were lyophilized and weighed again (final dry weight). The degree of enzymatic degradation of the samples was quantified as the percent weight loss (*W*%), which is calculated using the following formula:

*W*% = (*W*_0_-*W*_t_) / *W*_0_× 100%,

Where *W*_0_ represents the initial weight of samples and *W*_t_ represents the weight of corresponding sample after enzymatic degradation treatment.

**Hyaluronic acid degradation assay**

Samples were lyophilized and ground into powders and weighed (initial dry weight). Then, the samples (200 mg) were treated with hyaluronidase (1.5 mg/mL, Sigma Aldrich, Germany). After 24 h treatment, GAG content per dry weight was determined using the colorimetric assay (Genmed Scientifics Inc., USA) as described above.

***In vitro* antibacterial activity assays**

Staphylococcus aureus (*S. aureus*, Gram positive bacteria) and Escherichia coli (*E. coli*, Gram negative bacteria) was cultured using nutrient agar medium (Difco^™^, USA). The density of *S. aureus* was adjusted to 10^6^ colony forming units (CFU)/mL. ASB samples (~ 1 cm^2^) were sterilized by 75% alcohol, washed with PBS, and placed in the bottom of 24-well culture plate. Then, 1 mL bacterial suspension was added into each well of the plate to cover the ASB samples. Meanwhile, bacterial culture without ASBs was set as the positive control. The bacteria were further incubated at 37°C for 6 h with gentle shake. Afterward, a 100.0 µL bacterial suspension was taken and serially diluted to obtain a certain dilution, and then 100 μL of the diluted bacterial suspension was spread uniformly on a nutrient agar plate. After incubation at 37°C for 24 h, the number of bacterial colonies was counted and photographed [6]. In addition, 100 μL of the resuspended bacterial suspension was added to a test tube containing 10 mL of culture medium, and then the mixture was placed in a shaker (150 rpm) for 8 h. The absorbance was measured using a microplate reader (SpectraMaxM2e, molecular devices, USA) at 600 nm. The antibacterial rate was calculated using the following equation:

Antibacterial rate (%) = [(*OD*_con_-*OD*_test_) / *OD_con_* ] × 100%,

where *OD*_con_ and *OD*_test_, refer to the *OD*_600_ values of the control group and sample groups, respectively.

Biofilm inhibition assay was performed in a 24-well plate according to the method described previously [7,8]. ASB samples were prepared as described above and placed in a 24-well plate. Afterward, 1 mL bacterial suspension (10^6^ CFU/mL) was added into each well of plate to cover the ASB samples and incubated in an incubator (150 rpm). Bacterial culture without ASBs was set as the positive control. After being incubated at 37°C for 6 h, the surviving bacteria were resuspended with 1 mL of sterilized PBS. Then, 500 µL bacterial suspension was pipetted into a 24-well plate, and incubated for 72 h. The formed biofilm was washed two times with PBS and then stained with 100 µL of crystal violet (1 mg/mL) for 20 min. The biofilm was washed two more times with PBS and then photographed. Finally, the biofilm was dissolved in 100 µL ethanol (95%), and the absorbance at 570 nm was measured by using the microplate reader. The biofilm inhibition rate was calculated using the following equation:

Biofilm inhibition rate (%) = [(*OD*_con_-*OD*_test_) / *OD_con_* ] × 100%,

where *OD*_con_ and *OD*_test_, refer to the *OD*_570_ values of the control group and sample groups, respectively.

**Protein adsorption**

The anti-protein adsorption performance of the tested samples was evaluated using FITC-labelled bovine serum albumin (FITC-BSA) as the model protein. In brief, the samples (1 cm×1 cm) were placed in a 24-well plate and incubated with 500 μL FITC-BSA (1 mg/mL, Solarbio, China) at 37°C for 2 h. Then, the samples were rinsed with PBS three times and observed with fluorescence microscope.

**Blood experiments**

All the blood experiments were authorized by the Ethics Review Committee for Animal Experimentation of Changhai Hospital. Whole blood from healthy New Zealand white rabbits was used in blood experiments.

**Hemolysis assay**

Briefly, red blood cells (RBCs) were isolated from rabbit blood, washed and resuspended in PBS with the volume concentration of 2%. Samples (~1 cm^2^) were placed in the sterile eppendorf tube and 1 mL diluted RBC solution was added per tube and incubated at 37°C for 3 h. The RBCs incubated in deionized water and PBS were used as the positive and negative controls, respectively. After incubation, the supernatant was collected after centrifugation, and the optical absorbance of the solutions was recorded at 545 nm using a UV-Vis spectrophotometer. The hemolysis rate was calculated using the following equation:

Hemolysis rate (%) = (*OD*_test_-*OD*_neg_) / (*OD*_pos_-*OD*_neg_) × 100%,

where *OD*_test_, *OD*_neg_, and *OD*_pos_ were the *OD*_545_ values of samples, negative control and positive control, respectively.

**Preparation of platelet-rich plasma**

Platelet-rich plasma (PRP) was prepared as previously described [[9,10]](https://www.sciencedirect.com/science/article/pii/S0142961206003176#bib15). Briefly, rabbit blood was collected in tubes containing acid citrate dextrose anticoagulant and centrifuged at 300 g for 10 min and supernatant was removed. Plasma was isolated and centrifuged at 1200g for 10 min. Then, 85% volume of plasma was removed from the upper layer of centrifuge tube, and non-activated [PRP](https://www.sciencedirect.com/topics/medicine-and-dentistry/thrombocyte-rich-plasma) at the bottom of the centrifuge tube was collected.

**Platelet adhesion assay**

To observe platelet adhesion, the samples were prepared into 96-well plate-sized discs and incubated with the PRP for 1 h at 37°C under static conditions. The suspension was aspirated and each well was rinsed carefully three times with PBS. Subsequently, the morphology of adhered platelets on the surface of the samples was assessed via SEM. Quantitative analysis of platelet adhesion and activation was performed according to previous reports [11]. Briefly, adherent platelets were incubated with 2% Triton-PSB buffer for 30 min at 37°C to lyse platelets. Then, platelet adhesion on the sample surface was assessed by detecting the amount of lactate dehydrogenase (LDH) of platelet lysis by using ELISA kit (YOBIBIO, China).

**Complement activation assay**

The level of complement 3 (C3) activation was determined by using C3a ELISA kit (YOBIBIO, China). In brief, plasma was isolated by centrifugation from the whole blood of rats. Then, the tested samples (~1 cm^2^) were incubated with 500 µL plasma at 37°C. After incubation for 1 h, the plasma was collected and measured by using C3a ELISA kit according to the manufacturer's instructions.

***In vitro* closed-loop whole blood circulation model**

A closed-loop circulation was established according the previous studies [12,13]. It consisted of a roller pump, a thermostated water bath and a sample device, with closed silicone tubing circuits filled with 20 mL of rat blood. The thermostated water bath and a sample device were deployed with 50 mL polyvinyl chloride tubing (inner diameter of 2.5 mm). The closed-loop system was sterilized by circulating 70% alcohol overnight. After rinsing with sterile PBS, the inner surface of the loop system was then pre-coated with 1% bovine serum albumin (BSA) solution at 4°C overnight (Sigma, USA). The system was then rinsed with sterile saline solution and removed air bubbles. Fresh rabbit blood was collected in tubes containing acid citrate dextrose anticoagulant and then gently injected into the loop. After 1 h of circulation, the samples were taken out washed with PBS and cut into pieces for SEM analysis.

**Subcutaneous implantation in rats**

To evaluate the *in vivo* responses including biostability, cell ingrowth and calcification, the tested samples were implanted subcutaneously into Sprague–Dawley rats (150 ~ 200 g). In brief, rats were anesthetized, subcutaneous pockets were created on the dorsal side of each rat, and one small scaffold (~1 cm^2^) was inserted into each pocket. Subsequently, rats were allowed to recover and maintained in standard housing conditions with food and water ad libitum. At 30 days, rats were sacrificed and scaffold explants were harvested for histological analysis and calcium quantitative analysis.

***Ex vivo* arterio-venous shunt (AV shunt) assay in rabbits**

AV shunt assay was performed in New Zealand white rabbits (3.0–3.5 kg) to evaluate the *in vivo* antithrombotic effects of hydrogel coated ASBs according to the previous reports [14,15]. Briefly, an external circulation loop was constructed and the tested samples were tightly placed onto the inner wall of the circulating loop. After general anesthesia, the left carotid artery and the right jugular vein of the rabbits were isolated and connected with external circulation loop to form a closed-loop circuit. After 2 h interaction with the blood, the samples were taken out, rinsed with PBS and photographed. Subsequently, the samples were fixed with paraformaldehyde and the red blood cell adhesion on the test samples was observed by SEM.

***in situ* vascular implantation in rats**

The tested scaffolds were fabricated into small-diameter tubular grafts by rolling them up. Next, the constructed grafts were implanted *in vivo* to repair a segmental defect of rat carotid arteries following a cuff technique protocol [16,17]. In brief, the rats were anesthetized with 1% pentobarbital sodium (75 mg/kg of body weight), and then injected with heparin (250 units/kg) through the tail vein. After being completely unconscious, an incision at the midline of the neck was made and the left common carotid artery was isolated, clamped and transected. Then, the ASB tubular graft (1.0 mm in diameter and 6.0 mm in length) was sutured to the native artery with 8-0 suture by discontinuous end-to-end anastomosis. When blood flow was restored, the [surgical incision](https://www.sciencedirect.com/topics/medicine-and-dentistry/surgical-incision) was closed with 3-0 monofilament nylon sutures. No anticoagulation and antiplatelet drugs were administrated during the whole process.

**Histology**

The specimens were fixed in 4% buffered formaldehyde for 24 h, processed into paraffin, and then sectioned at 5 μm. Sections were deparaffinized and stained with hematoxylin and eosin (HE) for morphological examination, with Verhoeff van Gieson (EVG) staining for collagen and elastin, and with [alizarin](https://www.sciencedirect.com/topics/materials-science/alizarin) red S staining for detection of calcification. For EVG staining, sections were incubated in Verhoeff’s solution for 1 h, rinsed in water, differentiated in 95% ethanol, and incubated briefly in Van Gieson solution. For [alizarin](https://www.sciencedirect.com/topics/materials-science/alizarin) red S staining, deparaffinized tissue sections were incubated with 1% [alizarin](https://www.sciencedirect.com/topics/materials-science/alizarin) red S solution (pH 4.1). After incubation at room temperature for 20 min, the sections were washed again with deionized water to remove unincorporated dye.

**Immunohistochemistry**

Histological sections were deparaffinized and hydrated to distilled water. Sections then underwent antigen-retrieval in citrate buffer (0.01 M pH 6.0) at high temperature (water bath, 30 min at 98°C). After blocking for non-specific binding, primary antibody was applied at optimized concentrations and incubated overnight at 4°C. Subsequently, they were incubated with secondary antibody and streptavidin-peroxidase complex at room temperature for 15 min each (SP kit, Fujian Maixin, China), and visualized with 3,3'-diaminobenzidine (DAB, Fujian Maixin, China). Nuclear counterstaining was with haematoxylin. Primary antibodies included CD34 (Abcam, Cambridge, MA, dilution, 1:150) and α-smooth muscle actin (α-SMA) (Abcam, Cambridge, MA, dilution, 1:200).

**Supplementary Table 1. Characterization of various cardiovascular scaffolds**

| Material | Source | Biomechanics | Biocompatibility and calcification | Thrombogenicity and restenosis | Ref |
| --- | --- | --- | --- | --- | --- |
| ePTFE | Synthetic | -Microporous  -Fibril spacing ≈20–30 μm  -Relatively stiff  -Low compliance | -Cytotoxicity  -Inflammation  -Calcification | -High thrombosis risk  -[Restenosis](https://www.sciencedirect.com/topics/medicine-and-dentistry/restenosis) | 18,19 |
| Dacron | Synthetic | -High tensile strength and elastic modulus  -Resistant to degradation | -Early restenosis  -Inflammatory fibrosis  -Mismatch-related complications | -High thrombosis risk | 20 |
| Glut-crosslinked bovine pericardium | Xenogenic | -Increased tensile strength  -Increased stiffness  -Resistant to degradation | -Cytotoxicity  -Inflammation  -Immune reaction  -Severe calcification | -High thrombosis risk | 20,21 |
| PhotoFix | Xenogenic | -Degradation and remodeling  -Weak ultimate tensile  -Good elasticity | -Less cytotoxicity  -Severe inflammation  -Low calcification risk | -Improved hemocompatibility;  -Rapid endotheliazation | 22,23 |
| CardioCel | Xenogenic | -Degradation and remodeling  -Optimal tensile strength  -Excellent stiffness | -Less cytotoxicity  -Severe inflammation  -Low calcification risk | -Low thrombosis risk | 23,24 |
| CorMatrix | Xenogenic | -Degradation and remodeling  -Weaker ultimate tensile  -Good elasticity | -Good biocompatibility  -Non-immunogenicity  -Low inflammation | -High thrombosis risk | 25 |

ePTFE: expanded polytetrafluoroethylene; PhotoFix: photo-oxidized bovine pericardium; CardioCel: ultra-low concentration of monomeric Glut crosslinked bovine pericardium; CorMatrix: decellularized porcine small intestinal submucosa.

**Supplementary Table 2. Characterization of various hydrogel coating on cardiovascular scaffolds**

| Hydrogel material | Substrate material | In vitro/in vivo findings | Mechanical properties | In vivo model | Ref |
| --- | --- | --- | --- | --- | --- |
| Gelatin and thrombin-responsive nanoparticles | Decellularized porcine pericardium | -Good biocompatibility  -Superior anticoagulation  -Reduced calcification  -Minimal inflammation  -Endothelialization | No impact on the mechanical properties of substrate | Rabbit jugular vein implantation models, and rabbit carotid artery implantation | 26 |
| Polyethylenimine (PEI) and poly(2-acrylamido-2-methyl-1-propanesulfonic acid) (PAMPS) | Polyethylene terephthalate (PET) | -Hemocompatibility  -Endothelialization  -Antithrombus formation | Enhanced mechanical properties  Long-term stability | Canine Model for left atrial appendage Occluder | 27 |
| An inner dopamine-modified HA hydrogel and an outer gelatin hydrogel | Polycaprolactone (PCL) fibrous scaffold | -Good biocompatibility,  -Superior anticoagulation,  -Endothelialization | Enhanced [tensile modulus](https://www.sciencedirect.com/topics/materials-science/elastic-moduli) and [elongation at break](https://www.sciencedirect.com/topics/engineering/elongation-at-break) | N/A | 28 |
| Poly(carboxybetaine) microgel (pCBM) and poly(carboxybetaine) microgel (pCBM) | Polyvinyl chloride (PVC) substrate | -Good biocompatibility  -Low inflammatory response  -Antithrombotic properties | Enhanced mechanical properties  High stability | N/A | 29 |
| 3-sulfopropyl [methacrylate](https://www.sciencedirect.com/topics/pharmacology-toxicology-and-pharmaceutical-science/methacrylate) [potassium](https://www.sciencedirect.com/topics/materials-science/potassium) (SPM) and [acrylic acid](https://www.sciencedirect.com/topics/medicine-and-dentistry/acrylic-acid) and tert-butyl [peroxide](https://www.sciencedirect.com/topics/materials-science/peroxide) carbonate-2-ethylhexyl ester (TBEC) | Polyurethane (PU) | -Antithrombus  -Antiinflammation  -[Antioxidant activity](https://www.sciencedirect.com/topics/medicine-and-dentistry/antioxidant-capacity)  -H[istocompatibility](https://www.sciencedirect.com/topics/medicine-and-dentistry/histocompatibility)  -Anticalcification | No impact on the mechanical properties of substrate | Rabbit and pig vascular models | 30 |

**References**

1. Liu X, Wu H, Lu F, Li Q, Xu Z. Fabrication of porous bovine pericardium scaffolds incorporated with bFGF for tissue engineering applications. *Xenotransplantation* 2020;27:e12568.

2. Jiang W, Xiang X, Song M, Shen J, Shi Z, Huang W, Liu H. An all-silk-derived bilayer hydrogel for osteochondral tissue engineering. *Mater Today Bio* 2022;17:100485.

3. Munnelly AE, Cochrane L, Leong J, Vyavahare NR. Porcine vena cava as an alternative to bovine pericardium in bioprosthetic percutaneous heart valves. *Biomaterials* 2012;33:1-8.

4. Li P, Schille C, Schweizer E, Kimmerle-Müller E, Rupp F, Heiss A, Legner C, Klotz UE, Geis-Gerstorfer J, Scheideler L. Selection of extraction medium influences cytotoxicity of zinc and its alloys. *Acta Biomater* 2019;98:235-245.

5. Jin X, Wei C, Li K, Yin P, Wu C, Zhang W. Polyphenol-mediated hyaluronic acid/tannic acid hydrogel with short gelation time and high adhesion strength for accelerating wound healing. *Carbohydr Polym* 2024;342:122372.

6. Rezvan A, Sharifikolouei E, Lassnig A, Soprunyuk V, Gammer C, Spieckermann F, Schranz W, Najmi Z, Cochis A, Scalia AC, Rimondini L, Manfredi M, Eckert J, Sarac B. Antibacterial activity, cytocompatibility, and thermomechanical stability of Ti40Zr10Cu36Pd14 bulk metallic glass. *Mater Today Bio* 2022;16:100378.

7. Lv Y, Cai F, He Y, Li L, Huang Y, Yang J, Zheng Y, Shi X. Multi-crosslinked hydrogels with strong wet adhesion, self-healing, antibacterial property, reactive oxygen species scavenging activity, and on-demand removability for seawater-immersed wound healing. *Acta Biomater* 2023;159:95-110.

8. Ge X, Hu J, Qi X, Shi Y, Chen X, Xiang Y, Xu H, Li Y, Zhang Y, Shen J, Deng H. An Immunomodulatory Hydrogel Featuring Antibacterial and Reactive Oxygen Species Scavenging Properties for Treating Periodontitis in Diabetes. *Adv Mater* 2025;37:e2412240.

9. Wang K, Li J, Wang Y, Wang Y, Qin Y, Yang F, Zhang M, Zhu H, Li Z. Orchestrated cellular, biochemical, and biomechanical optimizations endow platelet-rich plasma-based engineered cartilage with structural and biomechanical recovery. *Bioact Mater* 2021;6:3824-3838.

10. Motlagh D, Yang J, Lui KY, Webb AR, Ameer GA. Hemocompatibility evaluation of poly(glycerol-sebacate) in vitro for vascular tissue engineering. *Biomaterials* 2006;27:4315-24.

11. Wang K, Li J, Wang Y, Wang Y, Qin Y, Yang F, Zhang M, Zhu H, Li Z. Orchestrated cellular, biochemical, and biomechanical optimizations endow platelet-rich plasma-based engineered cartilage with structural and biomechanical recovery. *Bioact Mater* 2021;6:3824-3838.

12. Wang D, Ge C, Liang W, Yang Q, Liu Q, Ma W, Shi L, Wu H, Zhang Y, Wu Z, Wei C, Huang L, Fang Z, Liu L, Bao S, Zhang H. In Vivo Enrichment and Elimination of Circulating Tumor Cells by Using a Black Phosphorus and Antibody Functionalized Intravenous Catheter. *Adv Sci (Weinh)* 2020;7:2000940.

13. Losi P, Lombardi S, Briganti E, Soldani G. Luminal surface microgeometry affects platelet adhesion in small-diameter synthetic grafts. *Biomaterials* 2004;25:4447-55.

14. Yu T, Pu H, Chen X, Kong Q, Chen C, Li G, Jiang Q, Wang Y. A versatile modification strategy for functional non-glutaraldehyde cross-linked bioprosthetic heart valves with enhanced anticoagulant, anticalcification and endothelialization properties. *Acta Biomater* 2023;160:45-58.

15. Hu M, Shi S, Peng X, Pu X, Yu X. A synergistic strategy of dual-crosslinking and loading intelligent nanogels for enhancing anti-coagulation, pro-endothelialization and anti-calcification properties in bioprosthetic heart valves. *Acta Biomater* 2023;171:466-481.

16. Fu J, Ding X, Stowell CET, Wu YL, Wang Y. Slow degrading poly(glycerol sebacate) derivatives improve vascular graft remodeling in a rat carotid artery interposition model. *Biomaterials* 2020;257:120251.

17. Schleimer K, Grommes J, Greiner A, Jalaie H, Kalder J, Langer S, Koeppel TA, Jacobs M, Kokozidou M. Training a sophisticated microsurgical technique: interposition of external jugular vein graft in the common carotid artery in rats. *J Vis Exp* 2012;(69):4124.

18. Losi P, Lombardi S, Briganti E, Soldani G. Luminal surface microgeometry affects platelet adhesion in small-diameter synthetic grafts. *Biomaterials*. 2004;25:4447-55.

19. Lejay A, Bratu B, Kuntz S, Neumann N, Heim F, Chakfé N. Calcification of Synthetic Vascular Grafts: A Systematic Review. *EJVES Vasc Forum*. 2023;60:1-7.

20. Orrapin S, Benyakorn T, Howard DP, Siribumrungwong B, Rerkasem K. Patches of different types for carotid patch angioplasty. *Cochrane Database Syst Rev*. 2021;2:CD000071.

21. Jiang Z, Wu Z, Deng D, Li J, Qi X, Song M, Liu Y, Wu Q, Xie X, Chen Z, Tang Z. Improved Cytocompatibility and Reduced Calcification of Glutaraldehyde-Crosslinked Bovine Pericardium by Modification With Glutathione. *Front Bioeng Biotechnol*. 2022;10:844010.

22. Carnagey J, Hern-Anderson D, Ranieri J, Schmidt CE. Rapid endothelialization of PhotoFix natural biomaterial vascular grafts. *J Biomed Mater Res B Appl Biomater*. 2003;65:171-9.

23. Neethling WML, Puls K, Rea A. Comparison of physical and biological properties of CardioCel® with commonly used bioscaffolds. *Interact Cardiovasc Thorac Surg*. 2018;26:985-992.

24. Patukale AA, Suna J, Anand A, Betts KS, Karl TR, Venugopal P, Marathe SP, Alphonso N. Performance of CardioCel in Cardiac Surgery: A Systematic Review. *World J Pediatr Congenit Heart Surg*. 2023;14:211-219.

25. Mosala Nezhad Z, Poncelet A, de Kerchove L, Gianello P, Fervaille C, El Khoury G. Small intestinal submucosa extracellular matrix (CorMatrix®) in cardiovascular surgery: a systematic review. *Interact Cardiovasc Thorac Surg*. 2016;22:839-50.

26. Pu H, Yu T, Xiong Y, Wang C, Zhou Z, Li G, Wang Y. Thrombin-regulated multi-functional hydrogel coating for decellularized extracellular matrix materials to enhance the anticoagulant and endothelialization properties. *Biomaterials*. 2025;326:123724.

27. Wang XW, Yin YJ, Wang J, Yu HM, Tang Q, Chen ZY, Fu GS, Ren KF, Ji J, Yu L. UV-Triggered Hydrogel Coating of the Double Network Polyelectrolytes for Enhanced Endothelialization. *Adv Sci (Weinh)*. 2024;11:e2401301.

28. Jiang Y, Guo Y, Wang H, Wang X, Li Q. Hydrogel coating based on dopamine-modified hyaluronic acid and gelatin with spatiotemporal drug release capacity for quick endothelialization and long-term anticoagulation. *Int J Biol Macromol*. 2023;230:123113.

29. Yao M, Wei Z, Li J, Guo Z, Yan Z, Sun X, Yu Q, Wu X, Yu C, Yao F, Feng S, Zhang H, Li J. Microgel reinforced zwitterionic hydrogel coating for blood-contacting biomedical devices. *Nat Commun*. 2022;13:5339.

30. Xiong Y, Hu Y, Wang J, Wang T, Hu J, He M, Wang L, Zhang Z, Lai W, Yang L, Luo R, Zhang F, Wang Y. In situ self-growth nano-selenium hydrogel coating alleviates surface thrombosis of blood-contacting devices by inactivating inflammatory cells. *Biomaterials*. 2026;324:123519.

**Supplementary Figures**


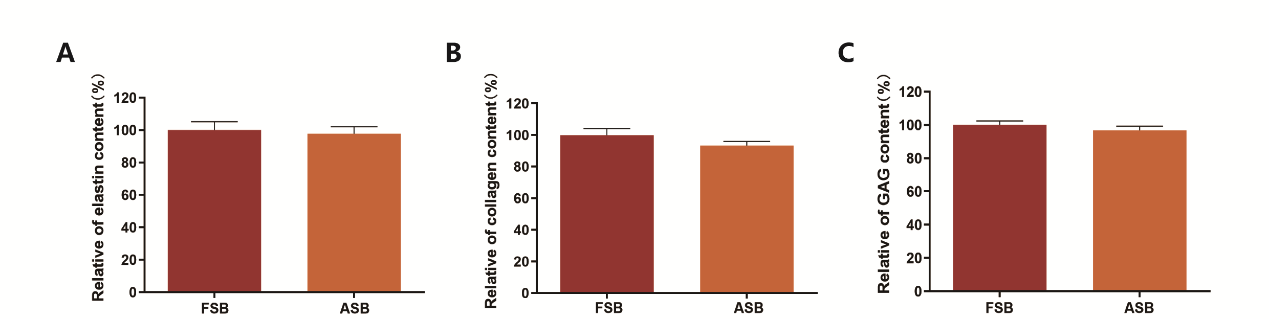


**Supplementary Figure 1. Comparison of (A) elastin, (B) collagen, and (C) GAG contents between FSBs and ASBs.**

**
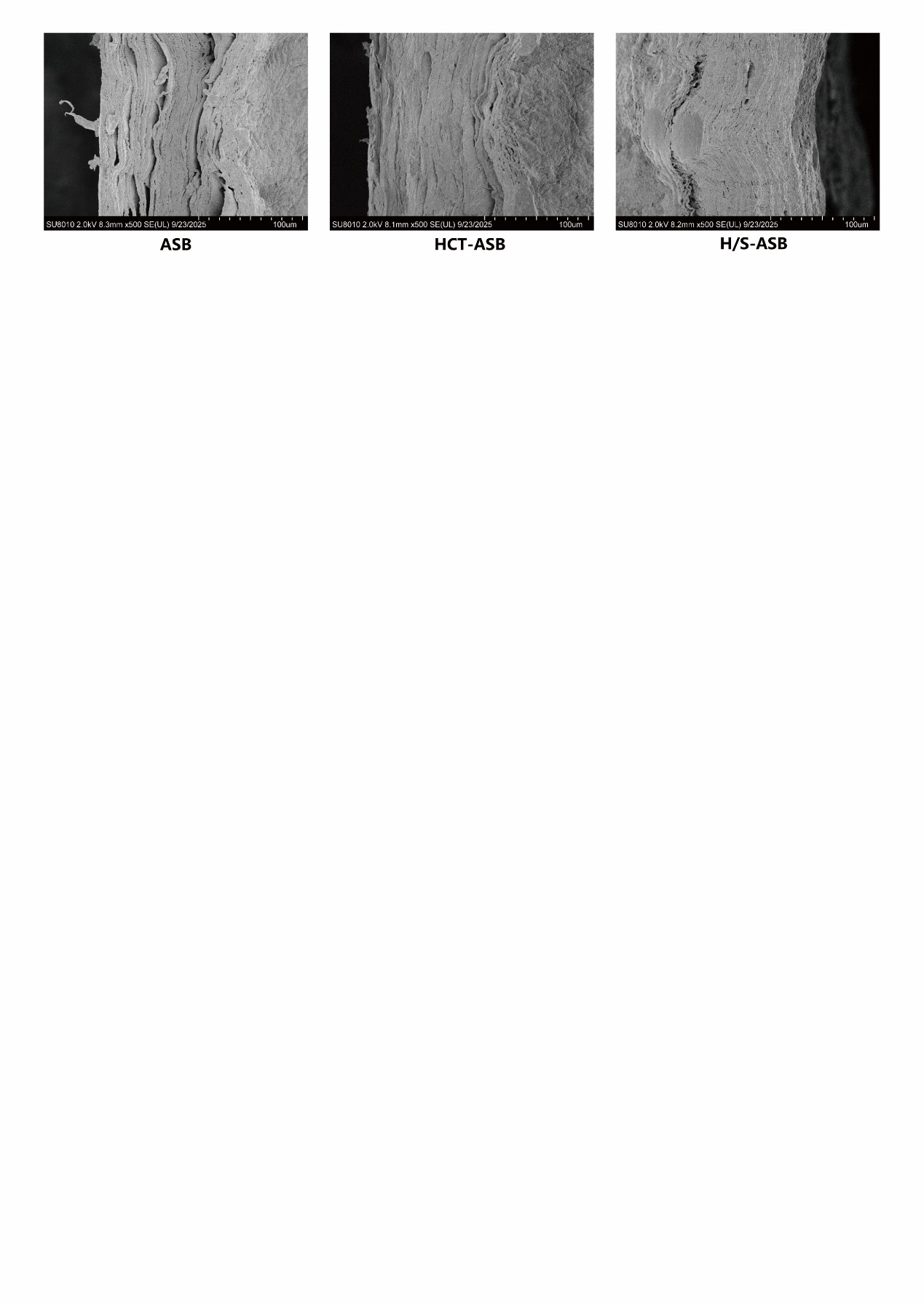
**

**Supplementary Figure 2. SEM images of the interface structure between the ASB scaffold and the bilayer hydrogel.**

**
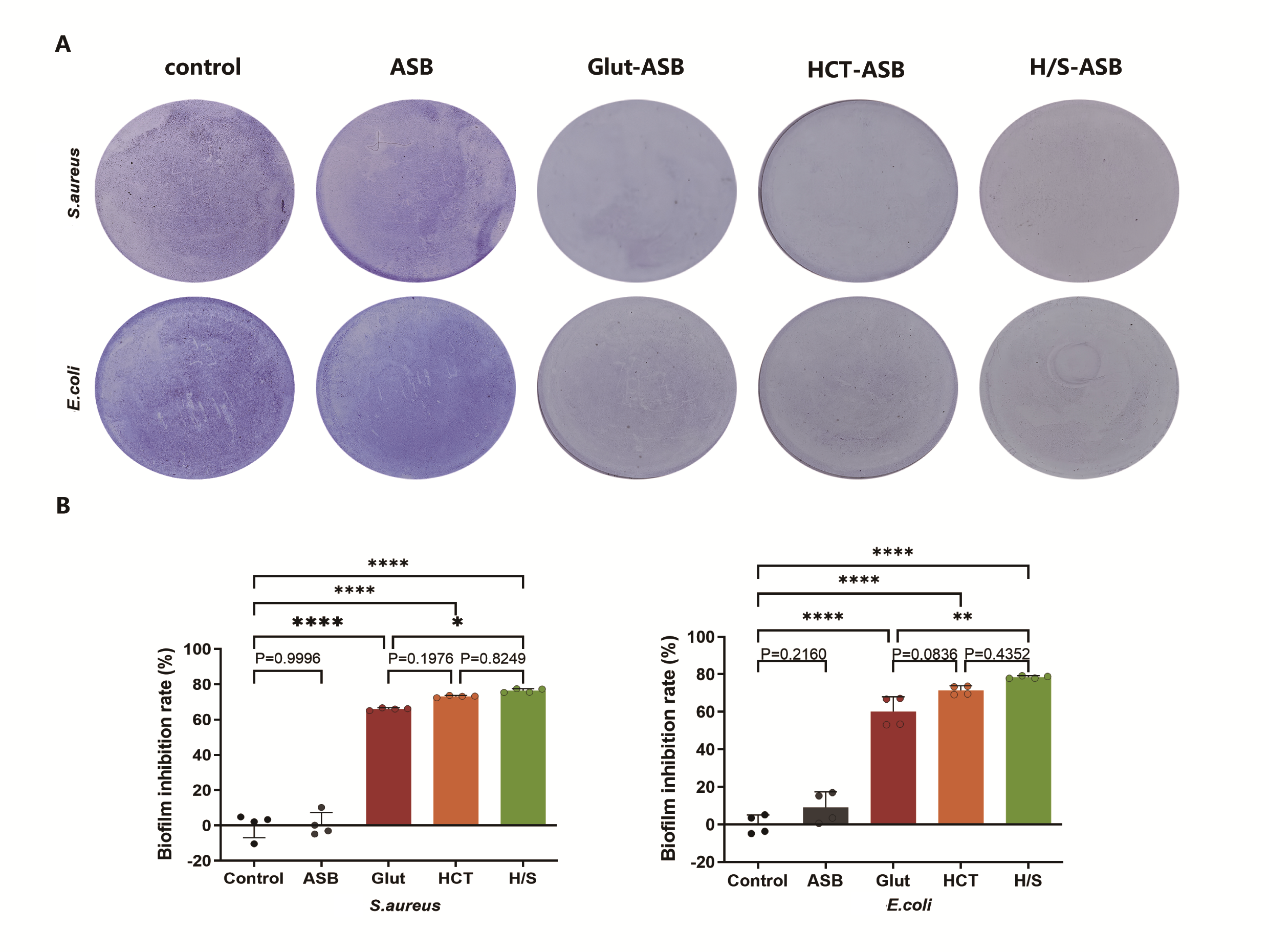
**

**Supplementary Figure 3. (A) Photographs and (B) quantification of biofilms stained with crystal violet following different treatments.**

**
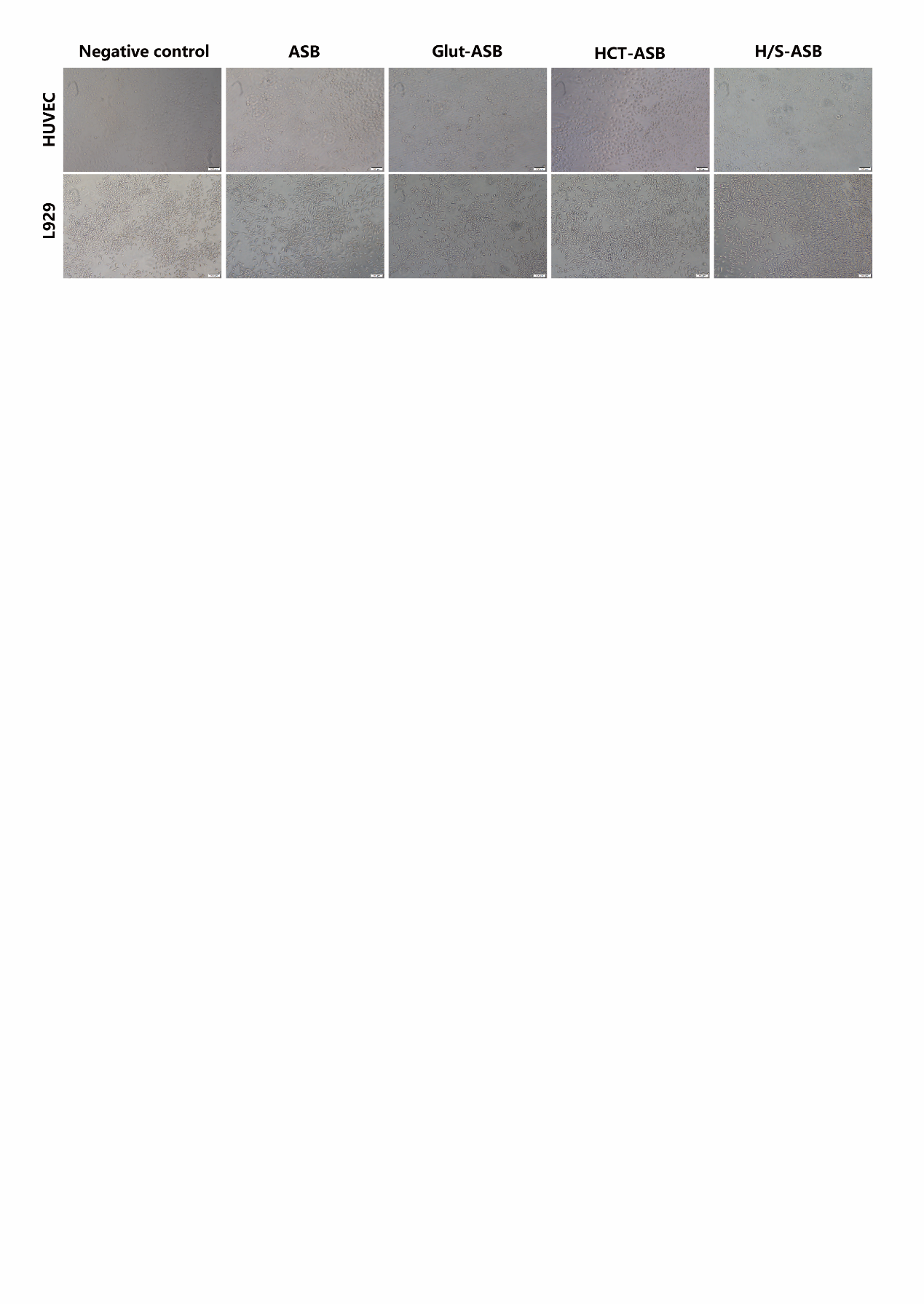
**

**Supplementary Figure 4. Cell morphology of HUVECs and L929 fibroblasts after incubation with ASB extract media for 3 days.** Scale bar = 100 µm.


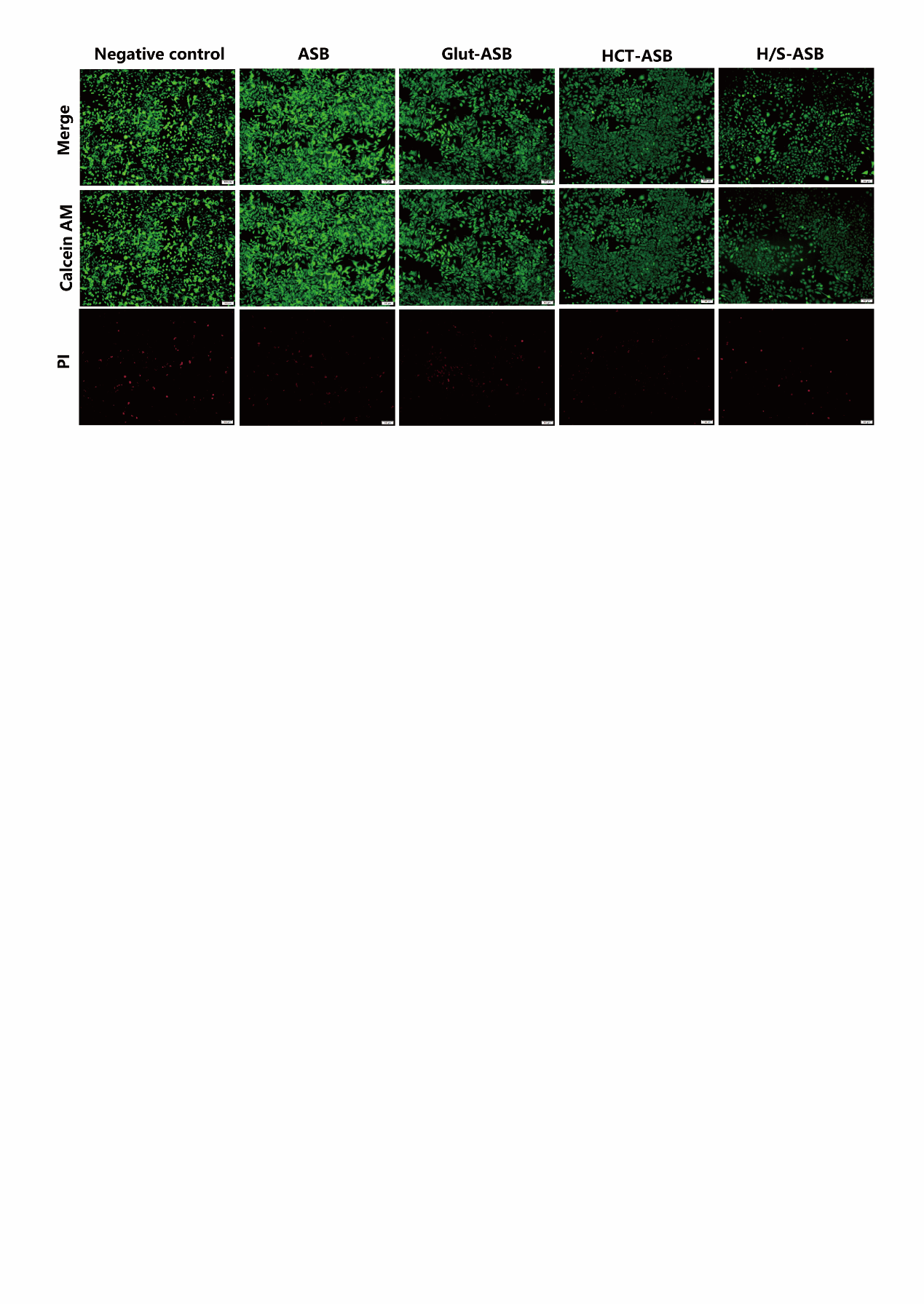


**Supplementary Figure 5. Cell viability of L929 cells was assessed using live/dead assay.** Scale bar = 100 µm.


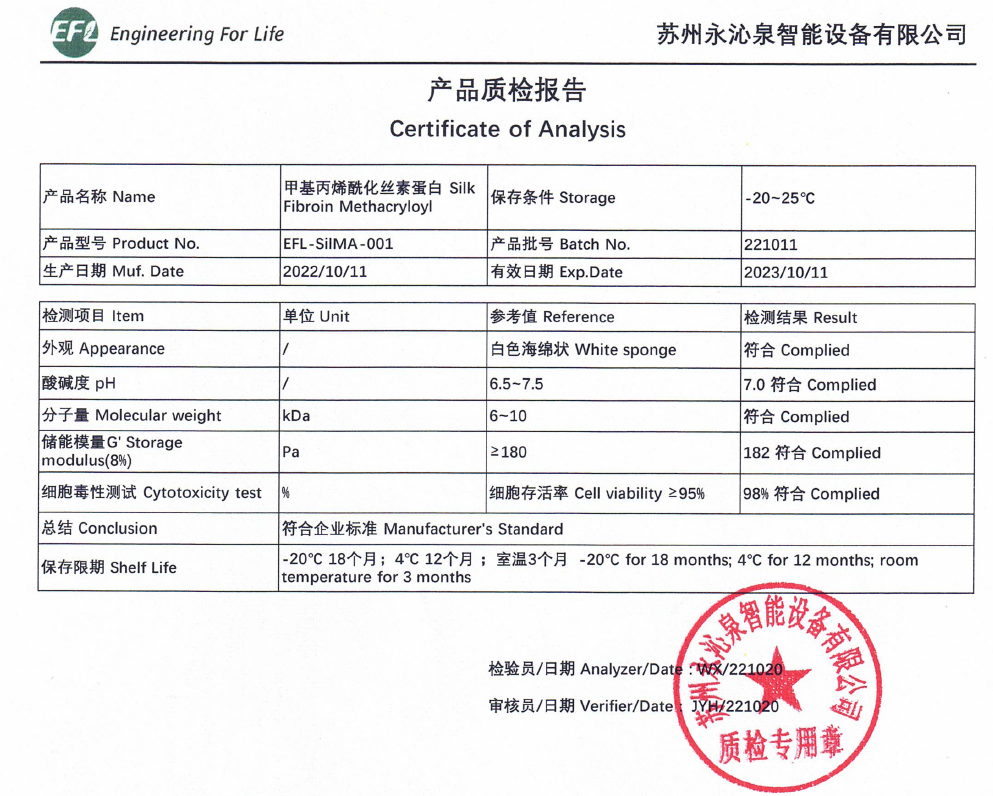


**Supplementary Figure 6. Identification report of SilMA.**
